# Rapid immune reconstitution following the infusion of autologous, Blinatumomab Expanded T-cells (BET) in patients with B-cell indolent NHL or CLL

**DOI:** 10.1038/s41408-024-01057-z

**Published:** 2024-04-26

**Authors:** Giuseppe Gritti, Silvia Ferrari, Federico Lussana, Anna Maria Barbui, Francesco Landi, Monica Rondi, Alessandro Putelli, Francesco Ballardini, Giulia Quaresmini, Muriel Paganessi, Chiara Pavoni, Arianna Ghirardi, Elisa Gotti, Chiara Capelli, Josée Golay, Martino Introna, Alessandro Rambaldi

**Affiliations:** 1 Hematology and BMT Unit, ASST Papa Giovanni XXIII, Bergamo, Italy; 2 Department of Oncology-Hematology, University of Milan, Milan, Italy; 3 Fondazione per la Ricerca Ospedale Maggiore (FROM), Bergamo, Italy; 4 Center of Cellular Therapy G. Lanzani, ASST Papa Giovanni XXIII, Bergamo, Italy

**Keywords:** Phase I trials, B-cell lymphoma, Chronic lymphocytic leukaemia

## Abstract

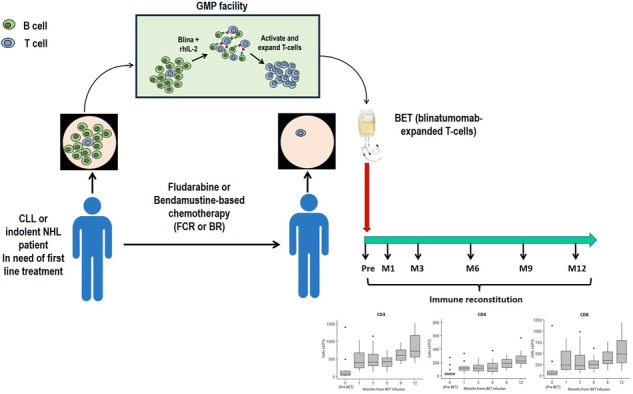

**TO THE EDITOR:**

First-line treatment with bendamustine-rituximab (BR) or fludarabine-cyclophosphamide-rituximab (FCR) is often used in indolent non-Hodgkin lymphoma (iNHL) and in selected cases of chronic lymphocytic leukemia (CLL) [1, 2]. Despite the good results, combination chemotherapy using drugs with purine analog or purine analogs-like moieties (i.e. fludarabine or bendamustine) results in sustained B and T-cell depletion that can last years after treatment [3, 4]. Indeed, it has been shown that efficient immunological recovery after chemotherapy requires multiple pathways that often evolve into a physiological age-dependent decline, resulting in a high frequency of long-term immune-deficient patients due to both dysfunctional and/or low absolute T-cell counts, especially in elderly patients [5].

Blinatumomab-expanded T cells (BET) are autologous polyclonal activated T cells expanded in vitro using the CD19xCD3 bispecific T-cell engager blinatumomab and rhIL-2 [6]. The use of blinatumomab, differently from the traditional co-stimulation with anti-CD3/CD28 beads, can both activate and expand T-cells while obtaining a rapid and complete clearance of normal and neoplastic B-cells. Thus, BET cells can be expanded from the peripheral blood (PB) of CLL and iNHL patients before treatment, preserving the normal T-cell polyclonality without contamination by neoplastic B cells [6].

We designed a phase I, dose escalation, open-label trial to evaluate the feasibility, safety and effect on immune recovery of BET cell infusion after first-line treatment with either FCR or BR in patients with iNHL and CLL (NCT03823365).

The trial design is summarized in Supplementary Fig. 1. BET cells were generated from 50 mL of PB before chemotherapy treatment, under good manufacturing practice (GMP) compliant conditions, as previously described [7].

Chemotherapy included 4 to 6 cycles of FCR or BR. Subjects with stable or progressive disease after the third cycle or suffering of serious treatment-related adverse events or with residual CD19^+^ B-cells (≥0.5%) in BET cell product were not eligible for cell infusion. BET was administered 2–5 days after the last chemotherapy. Dose escalation was conducted to assess the toxicity and efficiency of BET production. Four dose levels (3, 6, 9 and 12 × 10^9^ CD3^+^ cells) were evaluated with an accelerated titration design to assess the feasibility and toxicity of BET cells. In the expansion cohort, patients were infused with the whole cell product generated. Immune reconstitution parameters, including T cell subpopulation (CD4^+^ or CD8^+^ T regs, Th1/2/17, naïve, central and effector memory), NK-cell, B-cell and CMV-specific CD8^+^ cells were assessed at baseline (before chemotherapy), before BET infusion and after 1, 3, 6, 9 and 12 months. The polyclonality of CD3^+^ cells was assessed before chemo-immunotherapy, on BET cells product and 3 months after BET cells infusion.

A total of 19 patients were screened and 15 were found eligible. The CONSORT flow diagram is presented in Supplementary Fig. 2.

Clinical characteristics of the eligible patients are presented in Table 1. Eleven patients were diagnosed with CLL and 4 patients with iNHL, including 3 splenic marginal zone lymphoma (MZL) and one nodal MZL. Chemotherapy choice included FCR and BR in 5 and 10 patients, respectively. Twelve patients received the planned 6 cycles, while 2 patients received 4 cycles and one patient 3 cycles due to physician decision.

**Table 1:** Clinical characteristics.

	Total	CLL	iNHL
	*N* = 15	*N* = 11	*N* = 4
**Age, median (IQR)**	64.0 (62.0–69.0)	64.0 (55.0–71.0)	64.0 (62.0–67.5)
**Male Gender,** ***n*** **(%)**	12 (80.0)	8 (72.7)	4 (100.0)
**iNHL type,** ***n*** **(%)**			
*Splenic MZL*	-	-	3 (75.0)
*Nodal MZL*	-	-	1 (25.0)
**Ann Arbor stage (for iNHL),** ***n*** **(%)**			
*IV*	-	-	4 (100.0)
**Binet stage (for CLL),** ***n*** **(%)**			
*A*	-	5 (45.5)	
*B*	-	3 (27.3)	
*C*	-	3 (27.3)	
**Chemotherapy,** ***n*** **(%)**	15 (100.0)	11 (100.0)	4 (100.0)
*FCR*	5 (33.3)	5 (45.5)	0 (0.0)
*BR*	10 (66.7)	6 (54.5)	4 (100.0)
**Overall response,** ***n*** **(%)**			
*CR - Complete response*	8 (57.1)	7 (70.0)	1 (25.0)
*PR - Partial response*	6 (42.9)	3 (30.0)	3 (75.0)
**BET cell dose, (x10**^**9**^**)**			
*Median (range)*	9,5 (3–15.9)	10.41 (4.35–15.9)	5.04 (3–9)

A massive in vitro expansion of CD3^+^ cells and depletion of CD19^+^ cells were observed during BET production (Supplementary Fig. 3A and Supplementary Table 1). T-cell expansion was not correlated to B-cell contamination in starting material (Supplementary Fig. 3B). The polyclonality of CD3^+^ cells by flow cytometry of TCR Vß families was comparable on starting population of T cells and at the end of culture (Supplementary Fig. 3C). Expansion reduced the presence of T-regs in all the 15 patients and increased Th2 subset in most of the cases, while preserving the other CD4 and CD8 subpopulations (Supplementary Fig. 3D).

Fifteen patients received BET cells, 6 during dose escalation and 9 during expansion. Specifically, 4 patients received the planned doses of 3, 6, 9 and 12 × 10^9^ cells according to protocol, while 2 patients, due to insufficient cell production for assigned dose level, received the doses of 3 and 10.4 ×10^9^ cells, respectively. Nine additional patients were treated during dose expansion according to cell production, receiving a median dose of 9.6 ×10^9^ cells (range 4.35–15.9 × 10^9^ cells).

A total of 86 adverse events (AEs) occurring in 15 patients were reported during the trial, 27 (31%) of which occurred after BET cell infusion (Supplementary Table 2). Of note, no AE was reported attributable to BET cells. Four SAEs were reported during study, all related to infections and requiring hospitalization. There was one death during the study due to SARS-CoV-2 pneumonia occurring during COVID-19 pandemic.

After BET cells infusion, a rapid increase of T-cells was observed at month 1, persisting until month 6 (Fig. 1A). From month 9 a further growth of T-cell count was observed. Early increase was observed in both the CD4^+^ and CD8^+^ T-cell subsets (Fig. 1B, C). A non-significant trend towards better early T-cell immune reconstitution (at 1 and 3 months) was noted for patients infused with >9 × 10^9^ cells. This trend was mainly attributable to the CD8^+^ T-cell subset (Supplementary Fig. 4). The T cell subpopulations composition was preserved over time (Fig. 1D). Antiviral immunity for CMV could be assessed in 6 HLA-A2 positive patients, by CMV tetramer staining and flow cytometry. CMV-specific CD8^+^ cells boosted at month 1 and increased over time along with T-cell reconstitution (Supplementary Fig. 5). The polyclonality of CD3^+^ cells by flow cytometry of TCR Vß families was comparable before chemoimmunotherapy and after 3 months from BET infusion (Supplementary Fig. 6).

**Fig. 1:**
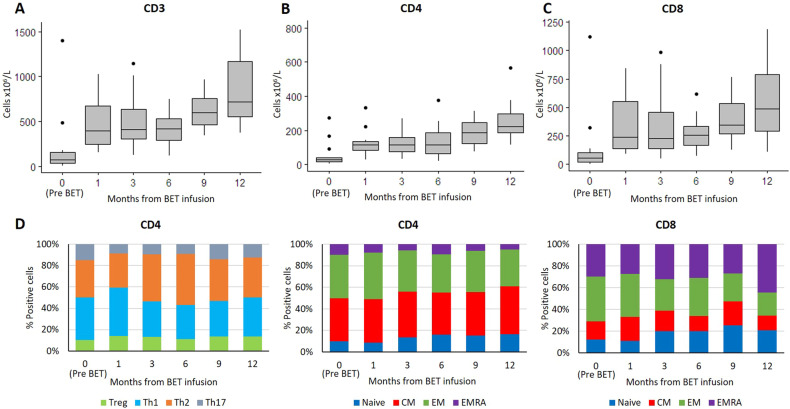
Immune reconstitution after FCR/BR chemotherapy and BET cell infusion. After infusion, a rapid increase of T-cells was observed after 1 month (**A**). Similar results were observed for CD4+ and CD8+ subsets, with a more prominent expansion of the latter (**B**, **C**). T cell subpopulation composition was preserved over time (**D**). *Legend: Treg: regulatory T-cells; Th1: T-helper 1 cells; Th2: T-helper 2 cells; Th17: T-helper 17 cells; Naïve: naïve T-cells; CM: central memory T-cells; EM: effector memory T-cells; EMRA: terminally differentiated effector memory T-cells re-expressing CD45RA*.

The current study evaluated for the first time a strategy that attempts to preserve T-cell function in patients with leukemic B-cell malignancies in need of lymphotoxic treatments, strategy that was previously hampered due to the invariable presence of clonal disease in cell products from iNHL/CLL patients.

We proved that BET cell production is clinically feasible starting from 50 mL of PB. Polyclonal T-cell profile as well as a maintenance of CMV^+^ CD8^+^ cells were preserved during the expansion process, confirming our previous preclinical findings [6]. Of note, Tregs, a subset of cell that contributes to immune impairment in CLL, were scarcely present in BET cell product [8].

BET cells could be administered without toxicity and leaded to a marked numerical increase of T-cells 1 month after infusion, suggesting that administration of ex vivo stimulated cells may promote immune reconstitution [3, 4, 9]. This early increase was noted for both the CD4^+^ and CD8^+^ T-cell subsets, and appeared more pronounced in those patients receiving a higher cell dose (>9 × 10^9^ cells). After the first month, a slowly progressive increase of T-cells was observed. Similarly, CMV-specific clones increased along time.

In previous reports, infusion of a higher dose (median of 43 × 10^9^ cells) of ex-vivo CD3/CD28 co-stimulated cells permitted to reach at early time points very high levels of circulating T-cells after autologous transplantation for multiple myeloma [10]. In our trial, we infused a lower number of cells and, similarly, we observed a rapid increase of T-cell at 1 month from infusion. It is possible that higher dose of BET cell therapy may be required to obtain stronger immune reconstitution.

Several confounding factors limit the interpretation of T-cell immune reconstitution in this study, including the specific diagnosis and age of the patient population, as well as the high complexity and variability of the immune reconstitution itself. Various infectious events were reported in this study both during chemotherapy and after BET cell infusion. Apart from the expected events during FCR and BR treatment, it should be noted that the trial was conducted across the COVID-19 pandemic, and SARS-CoV-2 pneumonia was fatal in one case. The design of the study and the low number of patients treated do not permit to evaluate if BET cellular therapy exerted a protective effect on infections. The trend in positive immune reconstitution with higher doses of BET warrant a clinical trial with high doses of these cells. The introduction of the G-Rex system that facilitates expansion of large number of BETs in smaller volumes should allow such trial to be performed in the future [11].

Apart from prevention of infections, the preservation of an intact T-cell functionality is currently of key importance in patients with B-cell malignancies, thanks to the advent of highly active immune-based treatments such as chimeric antigen-receptor (CAR) T-cells and T-cell redirecting bispecific antibodies [12–15]. The development of methods to preserve lymphocyte diversity and function may be relevant to promote the efficacy of subsequent immunotherapy. Last, but not least, the in vitro generation of BET cells may be used to rapidly obtain autologous, leukemia free-T cells for CAR-T cell production without neoplastic cells even when the starting lymphoid population is massively contaminated by tumor cells.

In conclusion, with this study we explored a novel modality to preserve the lymphoid functionality in patients with CLL and iNHL. We could confirm that BET cell treatment is safe and feasible in CLL and iNHL patients, leading to a meaningful early T-cell recovery.

### Supplementary information

Supplementary Figure Legend

Supplementary Figure 1

Supplementary Figure 2

Supplementary Figure 3

Supplementary Figure 4

Supplementary Figure 5

Supplementary Figure 6

Supplementary Table 1

Supplementary Table 2

Protocol

## Data Availability

Data supporting the findings of this study are available upon reasonable request from the corresponding authors. These data are not publicly available, and restrictions may apply according to the European General Data Protection Regulation (GDPR).
