# Supplementary material for: Rapid immune reconstitution following the infusion of autologous, Blinatumomab Expanded T-cells (BET) in patients with B-cell indolent NHL or CLL
Source: Blood Cancer J. 2024 Apr 26;14(1):73. doi: 10.1038/s41408-024-01057-z (PMC11053125; doi:10.1038/s41408-024-01057-z)
Supplement: Supplementary file 1 — Supplementary Figure Legend [file 41408_2024_1057_MOESM1_ESM.docx]

**Supplementary Figure legends**

**Supplementary Figure 1.** **Trial design:** Adult patients with untreated CLL/iNHL in need of first-line treatment with FCR or BR and absolute lymphocyte count >800x10^6^/L were eligible. BET cells were generated from 50 mL of peripheral blood before chemotherapy treatment and were infused after last chemotherapy cycle. Immune reconstitution parameters were assessed at study entry, before BET infusion and at definite time points during follow-up. Cell subsets were analyzed as previously reported (Golay et al., 2014): B-cells, T-cells (CD4^+^ and CD8^+^), NK cells, regulatory T-cells (Tregs), T-helper 1 (Th1), T-helper 2 (Th2) and T-helper 17 (Th17) CD4^+^ cells; CD8^+^ and CD4^+^ naïve, central memory (CM), effector memory (EM) and terminally differentiated effector memory re-expressing CD45RA (EMRA) T-cells (BD Bioscience, CA, USA). At the same time points, CMV-specific T cells were identified by flow cytometry after staining with PE-conjugated HLA-A*0201/pp65495–503 tetramer and anti-CD8 FITC (Immunotech Laboratories, Beckman Coulter, Marseille, France). Polyclonality of CD3^+^ cells was assessed by Vβ repertoire analysis using the Vβ repertoire kit from Beckman Coulter, CA, USA.

*Legend: BET: blinatumomab-expanded T-cell; iNHL: indolent non-Hodgkin lymphoma; CLL: chronic lymphocytic leukemia; PBMNC: peripheral blood mononuclear cell; FCR: fludarabine, cyclophosphamide, rituximab; BR: bendamustine, rituximab; M: month*

**Supplementary Figure 2.** **CONSORT diagram**

**Supplementary Figure 3.** **In vitro expansion of BET cells:** In vitro expansion of CD3^+^ cells and depletion of B-cell during BET production (**3A**). Correlation between absolute numbers of B-cell in starting material and T-cell expansion in terms of fold increase at the end of GMP culture (**3B**). Comparison of the polyclonality of CD3^+^ cells by flow cytometry of TCR Vβ families (**3C**) and T-cell subset composition (**3D**) on starting population of T cells and at the end of culture.

*Legend: Treg: regulatory T-cells; Th1: T-helper 1 cells; Th2: T-helper 2 cells; Th17: T-helper 17 cells; Naïve: naïve T-cells; CM: central memory T-cells; EM: effector memory T-cells; EMRA: terminally differentiated effector memory T-cells re-expressing CD45RA.*

**Supplementary Figure 4.** **Immune reconstitution after FCR/BR chemotherapy and BET cell infusion according to cell dose:** Comparison of CD3^+^ (panel A), CD4^+^ and CD8^+^ (panels B and C) cells for cell dose < or > 9x 10^9^ BET cells.

**Supplementary Figure 5**. **CMV-specific CD8^+^ cells after FCR/BR chemotherapy and BET cell infusion:** Percentage (**5A**) and absolute numbers (**5B**) of CMV-specific peripheral blood CD8^+^ cells from six HLA-A2+ patients positive for CMV.

**Supplementary Figure 6**. **TCR Vβ clonality after FCR/BR chemotherapy and BET cell infusion:** Comparison of the polyclonality of CD3^+^ cells by flow cytometry of TCR Vβ families before chemo-immunotherapy and after 3 months from BET infusion
