# Supplementary figures and images for: Rapid immune reconstitution following the infusion of autologous, Blinatumomab Expanded T-cells (BET) in patients with B-cell indolent NHL or CLL
Source: Blood Cancer J. 2024 Apr 26;14(1):73. doi: 10.1038/s41408-024-01057-z (PMC11053125; doi:10.1038/s41408-024-01057-z)

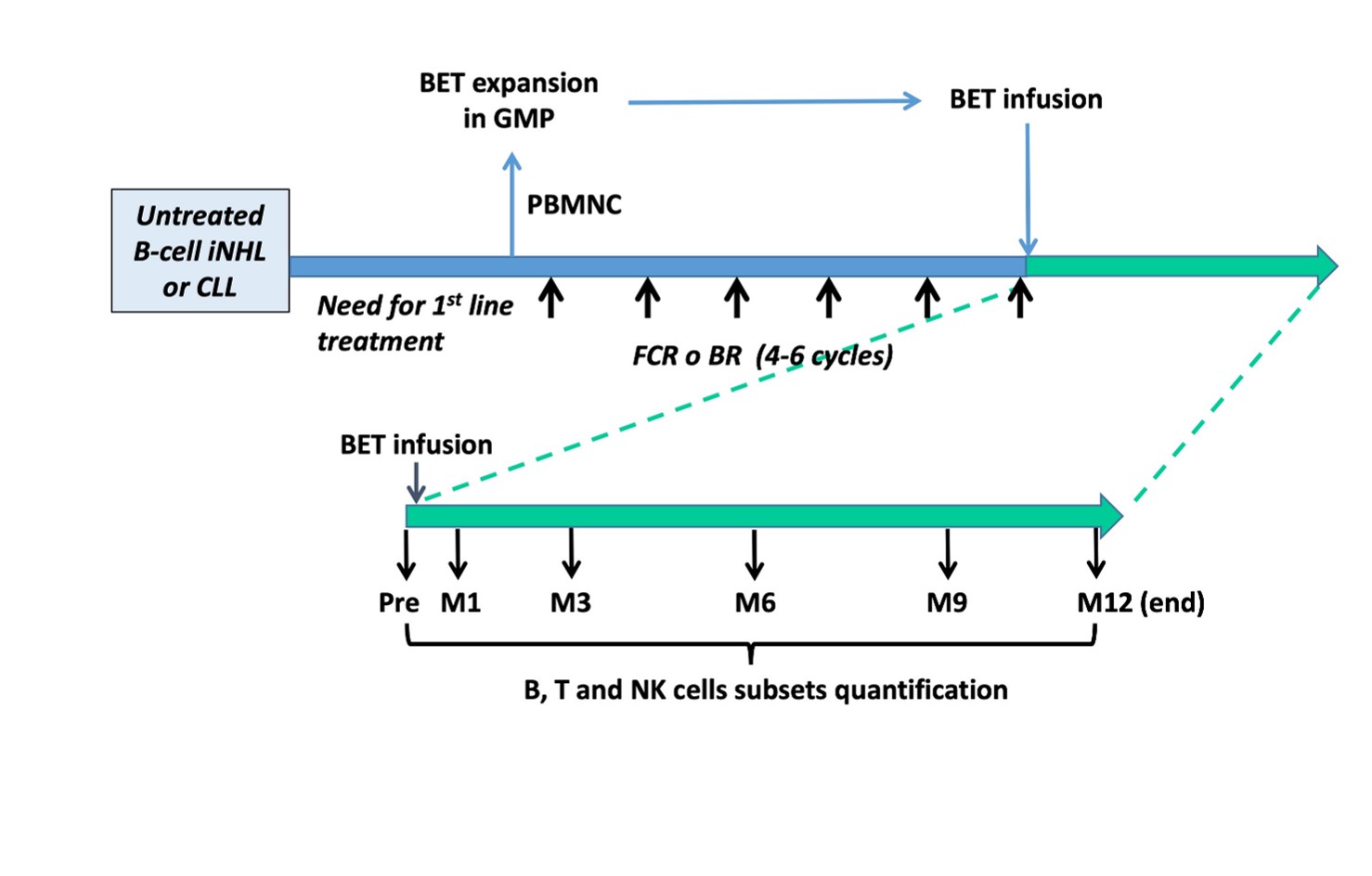

Supplement: Supplementary file 2 — Supplementary Figure 1 [file 41408_2024_1057_MOESM2_ESM.jpg]

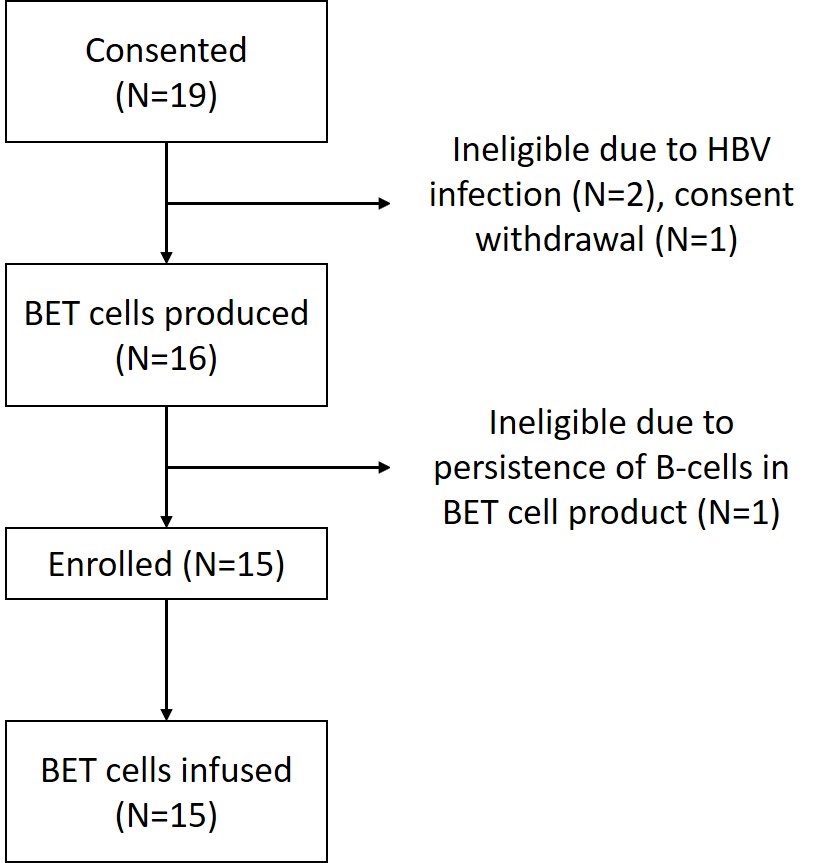

Supplement: Supplementary file 3 — Supplementary Figure 2 [file 41408_2024_1057_MOESM3_ESM.jpg]

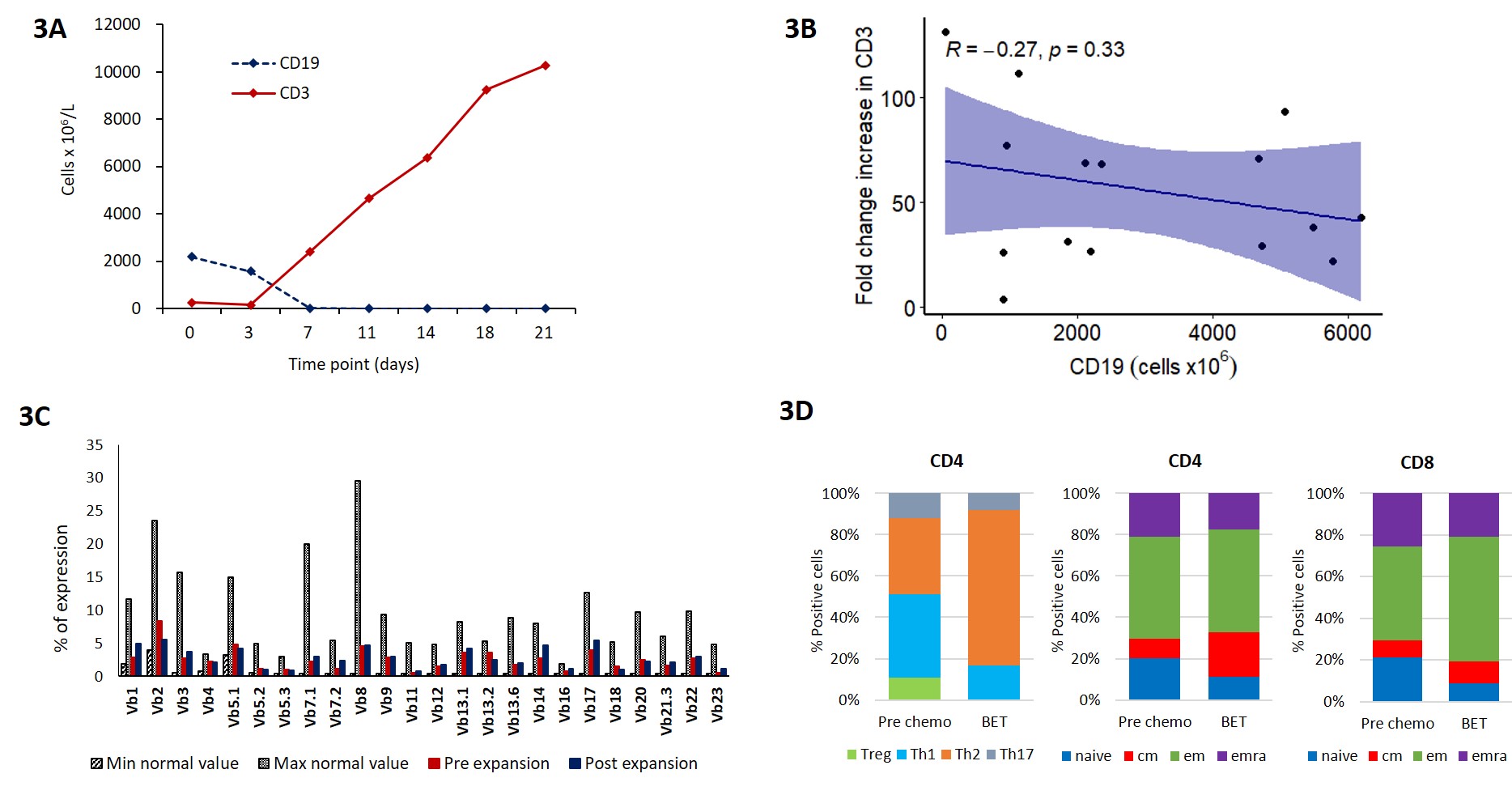

Supplement: Supplementary file 4 — Supplementary Figure 3 [file 41408_2024_1057_MOESM4_ESM.jpg]

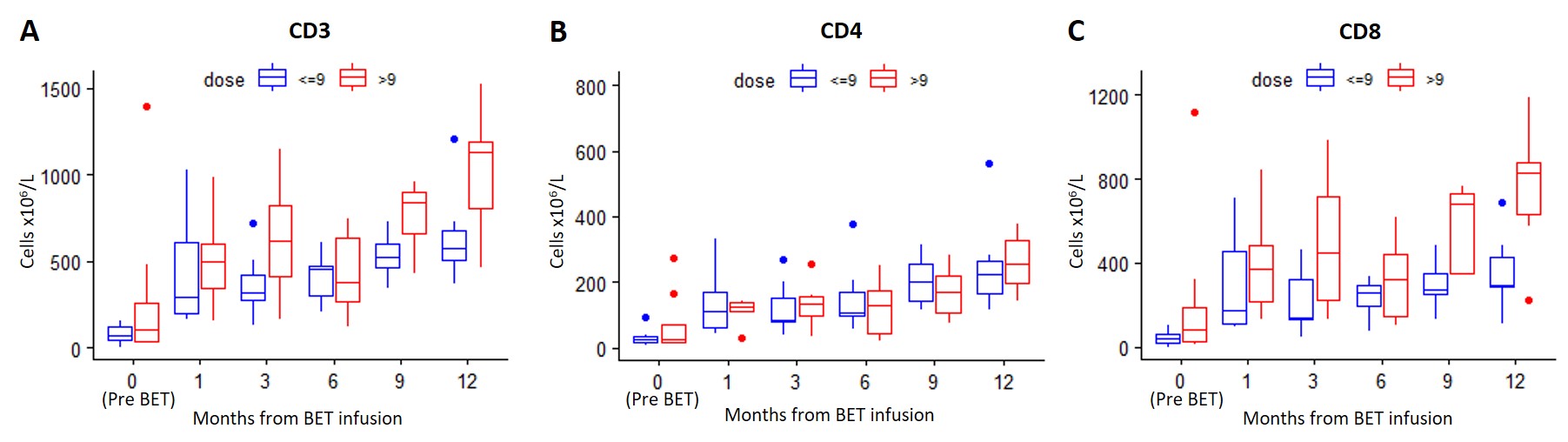

Supplement: Supplementary file 5 — Supplementary Figure 4 [file 41408_2024_1057_MOESM5_ESM.jpg]

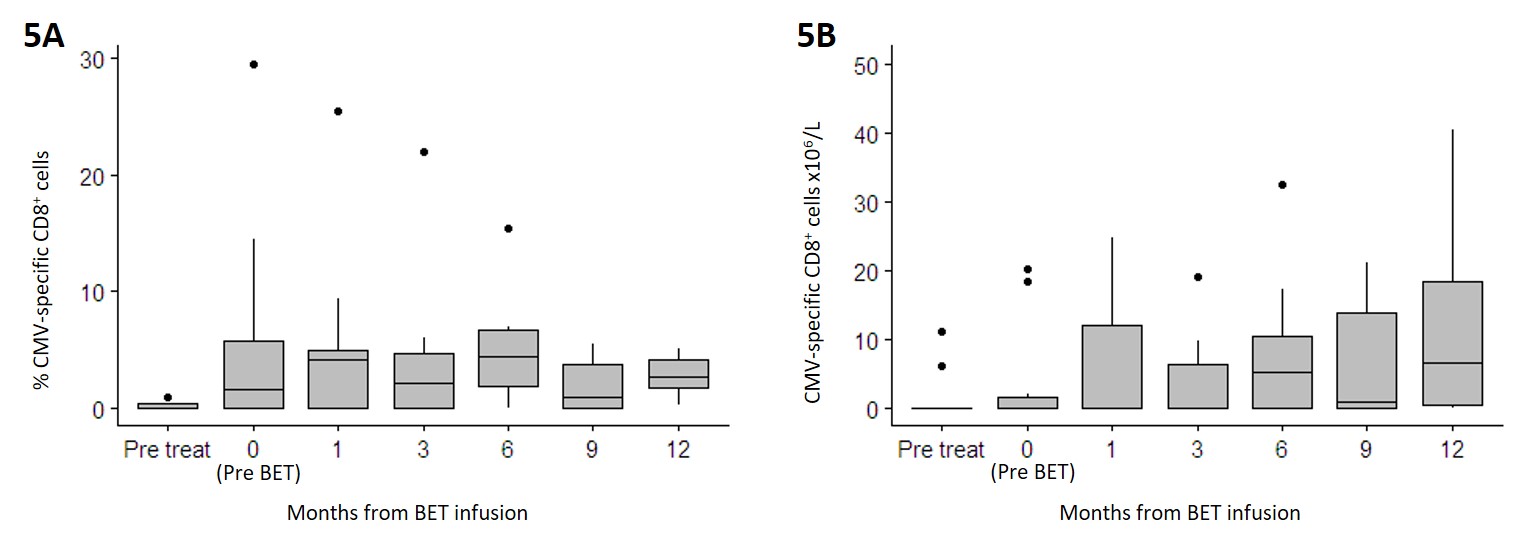

Supplement: Supplementary file 6 — Supplementary Figure 5 [file 41408_2024_1057_MOESM6_ESM.jpg]

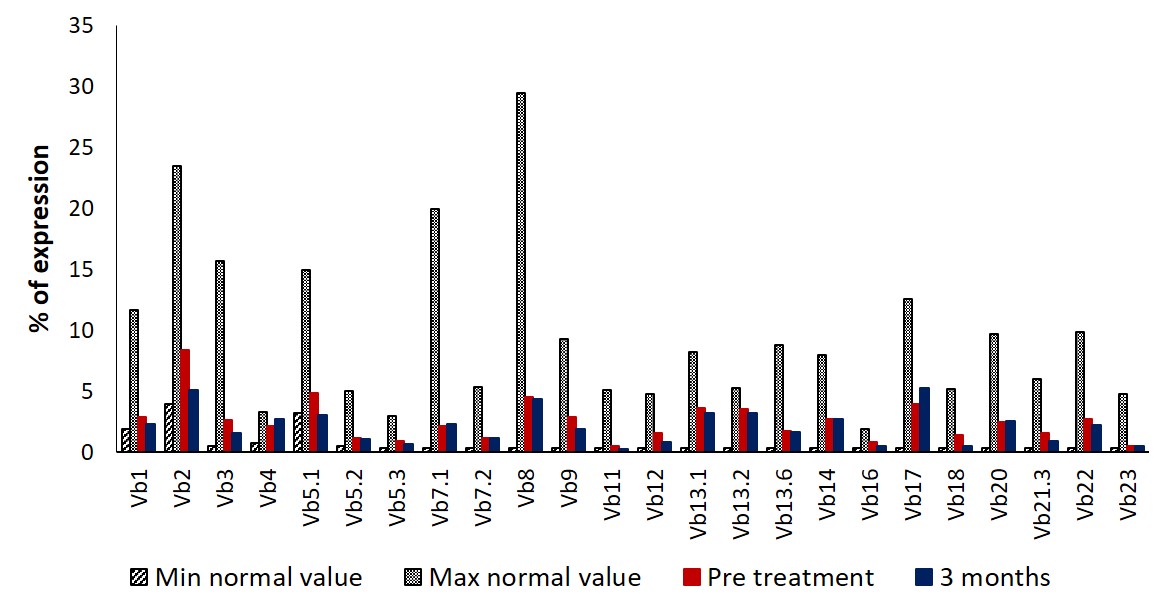

Supplement: Supplementary file 7 — Supplementary Figure 6 [file 41408_2024_1057_MOESM7_ESM.jpg]
