# Supplementary material for: Rapid immune reconstitution following the infusion of autologous, Blinatumomab Expanded T-cells (BET) in patients with B-cell indolent NHL or CLL
Source: Blood Cancer J. 2024 Apr 26;14(1):73. doi: 10.1038/s41408-024-01057-z (PMC11053125; doi:10.1038/s41408-024-01057-z)
Supplement: Supplementary file 8 — Supplementary Table 1 [file 41408_2024_1057_MOESM8_ESM.pdf]

Supplementary Table 2. Details of BET expansions

CELL EXPANSION																	GMP QUALITY CONTROLS (specifications)						Clinical Bags and Dose Levels		
	Input		Cells put in culture[a]					Days	Cell Product																
BET Lot ID	starting material	ml	TNC x10^6	%CD3$^+$	CD3$^+$ x10^6	%CD19$^+$	CD19+ x10^6	days	TNC x10^6	%CD3$^+$	CD3$^+$ x10^6	% CD4$^+$ (of CD3$^+$)	% CD8$^+$ (of CD3$^+$)	%CD19$^+$	% NK	Viability (%)	CD3$^+$ absolute number (>0,5x10^9)	Contaminating B cells (< 0,5%)	Viability (> 80%)	Sterility (sterile)	Endotoxin (< 7 EU/ml)	Mycoplasma (absent)	Total cryopreserved CD3$^+$ x10^9	Assigned dose level	CD3$^+$ infused cells x10^9
**7**	PB	44	2112	10,3	218	87,2	1842	18	6972	97,0	6763	13,4	80,5	0,0	2,0	83,0	√	√	√	√	√	√	6,0	1	3,0
**8**	PB	50	79	30,7	24	45,4	36	22	3384	94,2	3188	23,8	73,7	0,0	5,2	93,0	√	√	√	√	√	√	3,0	1	3,0
**9**	PB	44	2820	10,8	305	83,4	2352	22	21329	97,5	20796	20,2	73,7	0,0	1,0	86,0	√	√	√	√	√	√	20,4	2	6,0
**10**	PB	44	5250	2,3	121	96,3	5056	21	11890	94,9	11284	59,1	37,4	0,0	4,5	86,0	√	√	√	√	√	√	11,0	3	9,0
**11**	PB	42	2400	6,5	156	87,8	2107	21	11940	90,1	10758	19,2	77,4	0,0	9,0	93,0	√	√	√	√	√	√	10,4	3-4	10,4
**12**	PB	48	6795	10,1	686	85,0	5776	16	15104	98,8	14923	37,3	61,0	0,0	0,8	90,0	√	√	√	√	√	√	14,4	4	12,0
**13**	PB	47	4940	3,5	173	94,7	4678	21	12750	96,4	12291	69,5	28,9	0,0	1,8	80,0	√	√	√	√	√	√	12,0	expansion	12,0
**14**	PB	42	1040	5,7	59	90,2	938	24	4662	98,1	4573	24,5	72,3	0,0	1,6	85,0	√	√	√	√	√	√	4,4	expansion	4,4
**15**	DLI	105	5152	47,8	2463	17,3	891	21	9717	98,1	9532	55,4	38,5	0,0	1	92,4	√	√	√	√	√	√	9,0	expansion	9,0
**16**	PB	48	1280	21,8	279	70,1	897	22	7380	98,4	7262	19,6	78,2	0,0	1,5	89,7	√	√	√	√	√	√	7,1	expansion	7,1
**18**	PB	41	2678	13,6	364	81,8	2191	22	9800	99,1	9712	39,1	56,8	0,0	0,4	94,7	√	√	√	√	√	√	9,5	expansion	9,5
**19**	PB	43	5310	7,9	419	89	4726	20	12950	94,8	12277	72,7	19,5	0,0	4,7	98,9	√	√	√	√	√	√	12,0	expansion	12,0
**22**	PB	44	5840	4,4	257	93,8	5478	23	10556	92,1	9722	77,8	17,9	0,0	7,2	97,4	√	√	√	√	√	√	9,6	expansion	9,6
**23**	PB	47	6640	5,7	378	93,3	6195	21	16236	99,3	16122	38,8	58,7	0,0	0,3	89,9	√	√	√	√	√	√	15,9	expansion	12,0
**24**	PB	39	1320	8,3	110	85	1122	21	12397	98,4	12199	29,3	59,5	0,0	1,5	98,3	√	√	√	√	√	√	12,0	expansion	12,0
**17[b]**	PB	53	1160	34,4	399	45,3	525	21	1050	22,2	233	85,2	7,5	56,8	21,5	ND	No	No	No	ND	ND	ND	ND	ND	ND
	median[c]	*44,0*	*2820*	*8,3*	*257*	*87,2*	*2191*	*21,0*	*11890*	*97,5*	*10758*	*37,3*	*59,5*	*0,0*	*1,6*	*90,0*									
	min[c]	*39*	*79*	*2,3*	*24*	*17,3*	*36*	*16*	*3384*	*90,1*	*3188*	*13,4*	*17,9*	*0*	*0,3*	*80*									
	max[c]	*105*	*6795*	*47,8*	*2463*	*96,3*	*6195*	*24*	*21329*	*99,3*	*20796*	*77,8*	*80,5*	*0*	*9*	*98,9*									

[a] Post Ficoll and wash

[b] Lot which failed expansion

[c] Values calculated only from lots infused in patients (compliant)
