# Supplementary material for: Rapid immune reconstitution following the infusion of autologous, Blinatumomab Expanded T-cells (BET) in patients with B-cell indolent NHL or CLL
Source: Blood Cancer J. 2024 Apr 26;14(1):73. doi: 10.1038/s41408-024-01057-z (PMC11053125; doi:10.1038/s41408-024-01057-z)
Supplement: Supplementary file 9 — Supplementary Table 2 [file 41408_2024_1057_MOESM9_ESM.docx]

|  | **Total** | **Before BET infusion (during chemotherapy)** | | **First 6 months after BET infusion** | |
| --- | --- | --- | --- | --- | --- |
|  | **N (%)** | **Any grade** | **Grade >3** | **Any grade** | **Grade >3** |
| **AE** |  |  |  |  |  |
| Any | 86 | 59 | 13 | 27 | 6 |
| Related to BET | 0 (0%) | 0 (0%) | 0 (0%) | 0 (0%) | 0 (0%) |
| **Infections** | 20 (23.3%) | 10 (16.9%) | 0 (0%) | 10 (37%) | 3 (50.0%) |
| Pneumonia* | 3 (3.5%) | 0 (0%) | - | 3 (11.1%) | 2 (33.3%) |
| Bronchial infection | 6 (7%) | 5 (8.5%) | - | 1 (3.7%) | 0 (0%) |
| **SAE** |  |  |  |  |  |
| Any | 4 (4.7%) | 0 (0%) | 0 (0%) | 4 (14.8%) | 3 (50%) |
| Related to BET | 0 (0%) | 0 (0%) | 0 (0%) | 0 (0%) | 0 (0%) |
| *Including one grade 5 SARS-CoV-2 pneumonia | | | | | |

**Supplementary Table 2. Summary of adverse events**
