# Supplementary material for: Rapid immune reconstitution following the infusion of autologous, Blinatumomab Expanded T-cells (BET) in patients with B-cell indolent NHL or CLL
Source: Blood Cancer J. 2024 Apr 26;14(1):73. doi: 10.1038/s41408-024-01057-z (PMC11053125; doi:10.1038/s41408-024-01057-z)
Supplement: Supplementary file 10 — Protocol [file 41408_2024_1057_MOESM10_ESM.pdf]

# *Immune Reconstitution with Blinatumomab Expanded T-cells (BET) After First-line Treatment with Fludarabine-Cyclophosphamide-Rituximab or Bendamustine-Rituximab in CD20+ Indolent Non-Hodgkin Lymphomas/Chronic Lymphocytic Leukemia: a Phase I Study*

| | |
|---|---|
| IMP Identifiers: | *Blinatumomab Expanded T-cells (BET)* |
| Protocol Number: | *BET2017* |
| EudraCT Number: | *2018-000086-36* |
| Protocol to be amended: | *V 2; 17- May 2018* |
| Protocol Amendment NO. and Date: | *V 3; 15-September 2021* |
| Sponsor: | *ASST-Papa Giovanni XXIII* |

**CONFIDENTIAL**

This document contains confidential information belonging to Sponsor. Except as may be otherwise agreed to in writing, by accepting or reviewing these materials, you agree to hold such information in confidence and not to disclose it to others (except where required by applicable law), nor to use it for unauthorized purposes. In the event of actual or suspected breach of this obligation Sponsor should be promptly notified.

## SPONSOR SIGNATURE

Prof Alessandro Rambaldi
Sponsor Representative (printed name)

Signature | Date 21/DEC/2021

## STUDY CHAIR SIGNATURE *(on behalf of Steering Committee)*

Prof Alessandro Rambaldi
Principal Investigator (printed name)

Signature | Date 21/DEC/2021

## PRINCIPAL INVESTIGATOR AGREEMENT

I have read the Protocol entitled *"Immune Reconstitution with Blinatumomab Expanded T-cells (BET) After First-line Treatment with Fludarabine-Cyclophosphamide-Rituximab or Bendamustine-Rituximab in CD20+ Indolent Non-Hodgkin Lymphomas/Chronic Lymphocytic Leukemia: a Phase I Study"* and I agree to conduct the study as detailed herein and in compliance with ICH Guidelines for Good Clinical Practice and applicable regulatory requirements. I will provide all study personnel under my supervision with all information provided by the Sponsor and I will inform them about their responsibilities and obligations.

Prof Alessandro Rambaldi

UOC Ematologia
ASST- Papa Giovanni XXIII

Signature

21 DEC 2021
Date

# ADDITIONAL TRIAL PERSONNEL/SITE INFORMATION

| | |
|---|---|
| **Sponsor** | Name: ASST-Papa Giovanni XXIII<br>Address: Piazza OMS 1, 24127 Bergamo (BG) |
| **Principal Investigator** | Name/title: Prof Alessandro Rambaldi<br>Address: UOC Ematologia<br>ASST-Papa Giovanni XXIII, Piazza OMS 1, 24127 Bergamo (BG)<br>Phone: +39 035 2673683<br>Fax: +39 035 2674968<br>E-mail: arambaldi@asst-pg23.it |
| **Steering Committee** | Prof. Alessandro Rambaldi (Chairman), Dr. Giuseppe Gritti, Dr. Federico Lussana, Dr. Martino Introna, Dr Josée Golay. |
| **Safety desk (SAE Reporting)** | Name: PharmD Monia Lorini<br>UOC Farmacia<br>ASST-Papa Giovanni XXIII, Piazza OMS 1, 24127 Bergamo (BG)<br>Phone: +39 035 2673341<br>E-mail: mlorini@asst-pg23.it |

# TABLE OF CONTENTS

**TABLE OF CONTENTS** ..... **5**

**1 SYNOPSIS** ..... **8**

**2 STUDY FLOW CHART** ..... **19**

**3 SCHEDULE OF EVENTS** ..... **20**

**4 ABBREVIATIONS AND DEFINITIONS OF TERMS** ..... **22**

**5 BACKGROUND INFORMATION** ..... **25**

5.1 OVERVIEW OF DISEASE PATHOGENESIS, EPIDEMIOLOGY AND CURRENT TREATMENT ..... 25
5.2 INTRODUCTION TO INVESTIGATIONAL TREATMENT(S) AND OTHER STUDY TREATMENT(S) ..... 26
*5.2.1 Study drug* ..... *26*
*5.2.2 Nonclinical experience* ..... *28*
*5.2.3 Biological activity and pharmacodynamic data in applicable tumor models* ..... *28*
*5.2.4 Nonclinical risks* ..... *29*
*5.2.5 Clinical experience* ..... *30*

**6 STUDY RATIONALE** ..... **30**

6.1 STUDY RATIONALE AND PURPOSE ..... 30
6.2 RATIONALE FOR THE STUDY DESIGN ..... 32
6.3 RATIONALE FOR DOSE AND REGIMEN SELECTION ..... 32
6.4 RATIONALE FOR ENDPOINT DEFINITION ..... 33

**7 STUDY OBJECTIVES** ..... **35**

7.1 PRIMARY OBJECTIVE ..... 35
7.2 SECONDARY OBJECTIVE(S) ..... 35

**8 STUDY ENDPOINTS** ..... **36**

8.1 PRIMARY ENDPOINT ..... 36
8.2 SECONDARY ENDPOINT(S) ..... 36

**9 STUDY DESIGN** ..... **37**

9.1 OVERVIEW OF THE STUDY DESIGN ..... 37
9.2 CHOICE OF STARTING DOSE AND ESCALATED DOSE ..... 39
9.3 DOSE ESCALATION RULES: SEQUENCE AND TIMING OF PATIENTS INCLUSION ..... 39
9.4 STUDY DURATION AND DEFINITION OF END OF STUDY ..... 41
9.5 DOSE-LIMITING TOXICITIES (DLTS) AND INTERMEDIATE TOXICITY DEFINITIONS ..... 41
9.6 ESCALATION SCHEMA ..... 41
9.7 SEQUENCE OF PATIENTS ENROLLED ..... 43

**10 STUDY POPULATION** ..... **44**

10.1 SUBJECT SELECTION ..... 44
*10.1.1 Subject Inclusion Criteria* ..... *44*
*10.1.2 Subject Exclusion Criteria* ..... *46*
10.2 SCREENING FAILURES ..... 47
10.3 REPLACEMENTS ..... 47

**11 ENROLLMENT PROCEDURES** ..... **47**

**12 STUDY TREATMENT .......... 48**

12.1 TRIAL PRODUCT .......... 48
12.2 DRUG PREPARATION (IF NOT INCLUDED IN A SPECIFIC MANUAL) .......... 48
12.3 TREATMENT DOSE AND SCHEDULE .......... 49
12.4 DURATION OF TREATMENT AND ADMINISTRATION MANAGEMENT .......... 50
12.5 DRUG ACCOUNTABILITY .......... 51
12.6 TREATMENT DOSE MODIFICATIONS .......... 51
12.7 CONCOMITANT MEDICATIONS AND OTHER THERAPY .......... 51

**13 SUBJECT WITHDRAWAL FROM STUDY PARTICIPATION .......... 52**

**14 TREATMENT ASSESSMENT .......... 53**

14.1 PRE-TREATMENT EVALUATIONS .......... 53
14.2 ON STUDY EVALUATIONS .......... 55
14.4 FOLLOW-UP EVALUATIONS .......... 56
14.5 DETAILS OF INDIVIDUAL ASSESSMENTS .......... 57
*14.5.1 Safety endpoints assessment .......... 57*
*14.5.2 Efficacy endpoints assessment .......... 57*
*14.5.3 Other clinical evaluation, laboratory tests and follow-up .......... 57*
*14.5.4 Disease response assessment guidelines .......... 58*

**15 SAFETY ASSESSMENTS .......... 58**

15.1 PRE-EXISTING CONDITION .......... 58
15.2 ADVERSE EVENT ASSESSMENT .......... 59
15.3 ADVERSE EVENT REPORTING PERIOD .......... 60
15.4 REPORTING PROCEDURES FOR ADVERSE EVENT .......... 61
15.5 RECORDING ADVERSE EVENTS IN THE CASE REPORT FORMS .......... 62
15.6 CAUSALITY ASSESSMENT AND GRADING OF ADVERSE EVENT SEVERITY .......... 63
15.7 EXPOSURE IN UTERO .......... 64
15.8 OVERDOSE .......... 64
15.9 FOLLOW-UP OF UNRESOLVED ADVERSE EVENTS .......... 65

**16 EFFICACY ASSESSMENTS .......... 65**

16.1 DEFINITION OF EFFICACY PARAMETERS .......... 65

**17 STATISTICAL METHODS .......... 65**

17.1 SAMPLE SIZE CALCULATION .......... 65
17.2 STUDY POPULATION .......... 66
17.3 ANALYSIS .......... 66
*17.3.1 Study Conduct and Subject Disposition .......... 67*
*17.3.2 Baseline Characteristics and treatment Group Comparability .......... 67*
*17.3.3 Treatment Analysis .......... 67*
*17.3.4 Safety analysis .......... 67*
*17.3.5 Efficacy analysis .......... 68*

**18 QUALITY CONTROL AND QUALITY ASSURANCE .......... 68**

18.1 MONITORING .......... 69
18.2 AUDITING .......... 69
18.3 LABORATORY REQUIREMENTS .......... 69

**19 DATA HANDLING AND RECORD KEEPING .......... 69**

19.1 CASE REPORT FORM (CRF) .......... 69
19.2 DATA HANDLING .......... 70

19.3 RECORD RETENTION ... 70

**20 ETHICAL CONSIDERATION ... 72**

20.1 INSTITUTIONAL REVIEW BOARD(IRB)/ INDEPENDENT ETHICS COMMITTEE (IEC) AND COMPETENT AUTHORITY (CA) 72
20.2 ETHICAL CONDUCT OF THE TRIAL ... 72
20.3 INFORMED CONSENT ... 72

**21. STUDY DISCONTINUATION CRITERIA ... 73**

**22 LIABILITY AND INSURANCE ... 74**

**23 CONFIDENTIALITY OF INFORMATION AND PUBLICATION OF RESULTS ... 74**

**24 REFERENCES ... 76**

**25 APPENDICES ... 81**

APPENDIX A - ECOG PERFORMANCE STATUS SCALE ... 82
**APPENDIX B: CREATININE CLEARANCE CALCULATION: COCKCROFT-GAULT EQUATION** ... 83
**APPENDIX C: COMMON TERMINOLOGY CRITERIA FOR ADVERSE EVENTS** ... 84
**APPENDIX D: WORLD MEDICAL ASSOCIATION DECLARATION OF HELSINKI** ... 85

# 1 SYNOPSIS

| | |
|---|---|
| Title of Study: | Immune Reconstitution with Blinatumomab Expanded T-cells (BET) After First-line Treatment with Fludarabine-Cyclophosphamide-Rituximab or Bendamustine-Rituximab in CD20+ Indolent Non-Hodgkin Lymphomas/Chronic Lymphocytic Leukemia: a Phase I Study |
| Protocol Number: | *BET2017* |
| IMP: | • Blinatunomab Expanded T-cells (BET)<br>• Advanced Therapeutic Medicinal for somatic cell therapy<br>• Product Unit strength: cryopreserved sterile bags |
| Participating countries | Italy |
| List of study centres | ASST- Papa Giovanni XXIII (UOC Ematologia) |
| Background Information and Study Rationale | - Indolent non-Hodgkin lymphomas (iNHL) and chronic lymphocytic leukemia (CLL) are among the most frequent B-cell neoplasms (Ferlay et al., 2013). They include different histologies (i.e. follicular NHL, marginal zone NHL and lymphocytic NHL/CLL) characterized by chronic course and prolonged survival (Swerdlow et al., 2016). While some patients with limited stage disease may be cured, those presenting with advance stage or relapsing after local radiotherapy are generally considered not curable with standard treatments.<br>- First-line treatment of CLL/LL is currently based on the biologic profile of the disease (Robak et al., 2016). Excluding high risk patients harboring the del(17p) and/or TP53 mutations, first line chemoimmunotherapy options includes the use of either fludarabine, cyclophosphamide and rituximab (FCR) or BR. Despite the good results, treatment with FCR or BR regimens is associated with severe immunosuppression that worsens the immune dysfunctions already present at diagnosis in several patients. In the CLL phase III trial, high frequency of grade 3/4 infections was reported in FCR and BR, being observed in 39% and 25% of the patients, respectively (Eichhorst et al., 2016). In iNHL, infections have been observed in 37-55% of the patients treated with BR, with grade 3/4 events in 7-12% of the cases (Flinn et al., 2014; Rummel et al., 2013).<br>- Blinatumomab-expanded T cells (BET) are autologous polyclonal activated T cells expanded in vitro using blinatumomab and rhIL-2. They are an Advanced Therapeutic Medicinal Product (ATMP) for somatic cell therapy in the autologous setting. We have developed a method to expand and activate *ex vivo* the T lymphocytes present in the peripheral blood from CLL and iNHL patients for immunotherapeutic purposes. The traditional platform previously used worldwide to expand similar polyclonal T lymphocytes is centered on the concomitant addition of anti-CD3/anti- |

| | |
|---|---|
| | CD28 antibodies, bound to beads, to activate T cells through their TCR (CD3) and rhIL-2 to promote further T cells proliferation. This method works well when peripheral blood mononuclear cells (PBMC) of healthy donors is used, however, is not appropriate for T cell expansion from samples contaminated with neoplastic cells, because the procedure does not allow destruction of the latter, which remain at the end of culture. The Cell Factory Centro di Terapia Cellulare "G. Lanzani" assessed the functionality of our cell product in a mouse B-cell NHL xenograft model (Golay et al., 2014). Upon *in vivo* inoculation, BET retain functional activity: upon engagement with blinatumomab, BET were able to efficiently kill the B-cell NHL cells. BET therefore are able to recognize the tumor target through their TCR (by interaction with anti-CD3 portion of blinatumomab to TCR and CD19 on tumor cells), activate and kill the target tumor. These data therefore indicate that the infused BET maintained their full T cells functionality. Importantly, BET did not showed any toxicity in animals, even at high doses and in presence of blinatumomab.<br>Detailed information regarding the nonclinical pharmacology and toxicology of BET can be found in the Investigator's Brochure (IB) V1, 16/07/2017.<br>- About clinical experience, it has been previously shown that adoptive transfer of *ex vivo* (anti-CD3/anti-CD28) co-stimulated autologous T cells can successfully accelerate a robust T-cell recovery early after autologous transplant for multiple myeloma (Rapoport et al., 2005; Rapoport et al., 2009). However, the invariable presence of clonal disease in cell product of iNHL/CLL patients hampered this possibility up to now. The Cell Factory Centro di Terapia Cellulare "G. Lanzani" previously reported a simple, GMP-compliant protocol for *ex vivo* expansion of normal T cells from CLL patients peripheral blood for adoptive therapy (Golay et al., 2014). They showed that starting from only 10 mL of peripheral blood, a mean $5.15 \times 10^8$ CD3+ cells can be expanded in 3 weeks with a rapid clearance of CLL contamination. The resulting blinatumomab-expanded T cells (BET) were polyclonal CD4+ and CD8+ T cells and mostly effector and central memory cells. They showed a normalized expression of the synapse inhibitors CD272 and CD279 compared with starting T cells and were functionally active, showing cytotoxicity against CD19+ targets in presence of Blinatumomab *in vitro* and *in vivo*.<br>- On the basis of these data we hypothesize that BET infusion after first-line treatment of iNHL/CLL with either FCR or BR could lead to an adequate immune recovery. |
| Primary Objective | To determine the MTD, and safety profile of BET cells infusion |
| Secondary Objective(s) | <u>Secondary safety objectives</u><br>- To further define the safety and tolerability of BET cells infusion<br><br><u>Secondary efficacy objectives</u><br>- To evaluate the efficiency of BET cells expansion |

| | |
|---|---|
| | - To determine the optimal biological dose (OBD) of BET cells<br>- To characterize the general immune reconstitution after BET infusion<br>- Evaluation of ex vivo transfer of anti-viral immunity |
| Primary Endpoint | Assessment of Dose Limiting Toxicities (DLTs, defined as any grade 3 or 4 events that are considered by the investigator to be at least possibly related to therapy) observed during 14 days after BET infusion: four escalating dose cohorts will be evaluated and monitored for DLTs (and safety) in order to define MTD. |
| Secondary Endpoint(s) | Secondary safety endpoint<br>- Adverse event (AE) and laboratory abnormalities will be continuously assessed throughout the study and reported at each scheduled visit. Description and grading of all adverse events will be based on the NCI –CTCAE v4.03 and MedDra code (current version).<br><br>Secondary efficacy endpoint<br>- Absolute number of BET expanded and their composition (in terms of T-cell subsets and NK cells) in relation to absolute number of starting CD3+ and CD19+ lymphocytes<br>- Absolute CD3+ count +90 days after infusion in relation to the absolute number of BET cell infused and its composition (in terms of T-cell subsets and NK cells). OBD of BET will be defined as the absolute number of BET cell that will allow a CD3+ count of $\geq$ 600 x$10^6$/L at +90 days after infusion in at least 70% of the patients.<br>- Absolute numbers of B, T, and NK cells reconstitution at +0 (4 hours), +30, +90 and +180 days after infusion and its correlation with BET cell infused and its composition (in terms of T-cell subsets and NK cells)<br>- Evaluation of ex vivo transfer of anti-viral immunity in terms of tetramer-based quantification of CMV-specific CD8+ T lymphocytes at +0 (4 hours), +30, +90 and +180 days after infusion (this will be done only for CMV positive patients for whom CMV specific tetramers stain positive in starting peripheral blood or BET). |
| Study Design | This study is a phase I open-label, single center study. The patient population will consist of adults diagnosed with indolent non-Hodgkin lymphomas (iNHL) or chronic lymphocytic leukemia (CLL) in need of first line treatment consisting of either FCR or BR as per investigator assessment. About 48 mL of PB will be drawn by phlebotomy or apheresis before treatment and will be used to generate BET under GMP compliant conditions. Cell product will be evaluated for the presence of residual B cells by flow cytometry, patients with measurable ($\geq$0.5%) residual B-cells in the final BET product will be excluded from the study. Chemotherapy treatment will consist of a minimum of 4 to a maximum of 6 cycles of standard FCR or BR. Subjects with stable or progressive disease after the third cycle or suffering of serious treatment-related adverse events will not be eligible for the study. Patients will be |

enrolled in the study after the last planned cycle of chemo-immunotherapy and, if eligibility criteria are met, BET dose level will be assigned. Two (≥ 48 hours) to five days after the last chemotherapy infusion, BET will be administered according to the following schema.

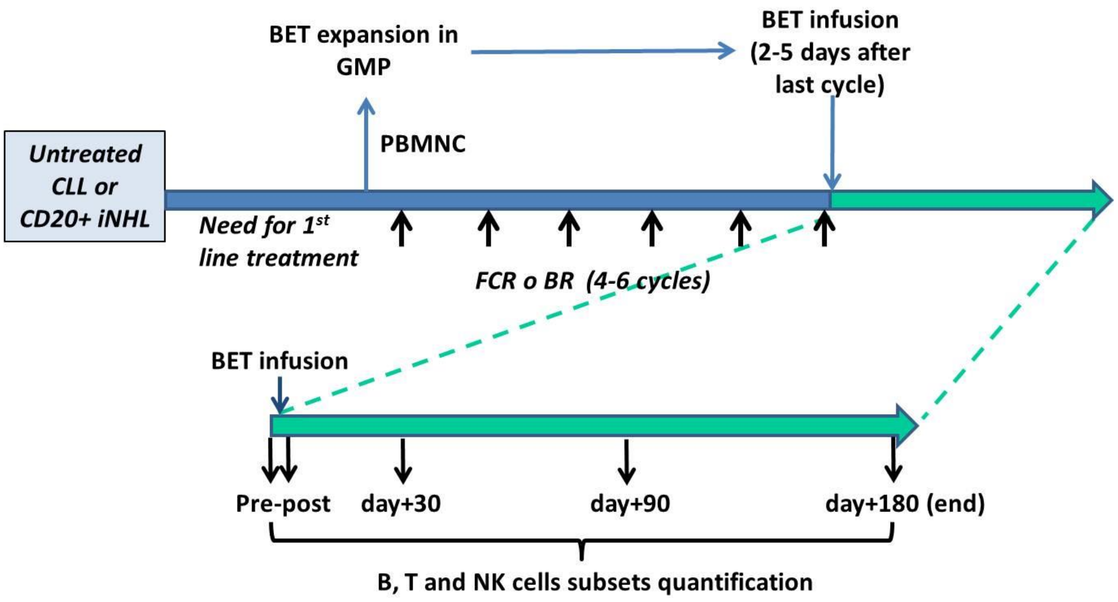


An accelerated titration dose escalation design will be used, followed by a dose expansion cohort of 9 patients. During dose escalation, up to four dose levels (table below) will be evaluated or until MTD is reached, as summarized in the Flow Chart of patients allocation. As the expansion rate is variable due to the highly variability of starting material in terms of number and percentages of T cells, in the case of insufficient BET production for the assigned dose level, the patient will be treated at the lower dose or at an intermediate dose level and will be replaced. In case the number of cells obtained are not sufficient to administer to the patient the starting dose level of 3 x $10^9$, the minimum of 0.5x$10^9$ CD3+ is accepted as the minimum dose for treatment; if it is not reached the patient will be considered as failing screening and excluded from participation to BET trial.

| *Dose level* | *BET dose (Counted on CD3+ cells)* |
|---|---|
| 1 (starting dose) | 3.0 x $10^9$ |
| 2 | 6.0 x $10^9$ |
| 3 | 9.0 x $10^9$ |
| 4 | 12.0 x $10^9$ |

| | |
|---|---|
| Treatment | • Two (i.e. ≥48 hours) to five days after the last chemotherapy infusion (in order to allow clearance of cytotoxic drug before BET infusion), BET will be administered. During dose escalation, cells may be frozen in up to 3 bags at the following amounts: 3.0x10$^9$, 3.0x10$^9$, and 6.0x10$^9$ BET cells per bag. The assigned dose level (table above) will be administered as 1, 2 or 3 bags (max 100 ml).<br>• The pharmacodynamics of BET have been studied in vivo in a mouse model of primary diffuse large cell lymphoma (DLBCL). BET were inoculated in very large doses (20x10$^6$/mouse /inoculum) i.v. every 14 days, for a total of 4 treatments, and have shown anti-tumoral activity in presence of blinatumomab without any detectable toxicity.<br>Similar cell product (i.e. activated T-lymphocytes) have been previously infused in humans by the group of Lanzani's Laboratory (Introna et al., 2007; Introna et al., 2010) and others (Rapoport et al., 2005; Rapoport et al., 2009), and no significant toxicities have been reported, with the exception of mild infusion. Aiming to limit the proportion of patients treated at subtherapeutic dose levels and reducing trial duration, a accelerated titration dose escalation design will be used to determine the maximum tolerated dose (MTD) of BET over 4 dose levels.<br>• Based on large-scale validation experiments performed at Lanzani's Cell Factory, the mean production of CD3+ T cells (BET) starting from 21 ml of whole blood was 3.6 x10$^9$ (range: 0.9-9.4 x10$^9$). Accordingly, in this protocol we will assess the effect of BET infusion on lymphoid immune reconstitution with a starting dose level in the range of the number of the non-stimulated T-cell usually infused during autologous SCT (3.0 x10$^9$), up to the maximum reasonably reachable with the expansion procedure (12.0 x 10$^9$). Thus, the dose level of BET cells planned to be assessed in the present study have been calculated basing on both scientific data and the expected production potential of the Cell Factory. |
| Escalation Scheme | Intended dose levels: (see table below)<br>Dose escalation rules: (see table below) |

Intended dose levels:

| Dose Level | BET dose (±10%) (*Counted on CD3+ cells*) |
|---|---|
| Level 1 | 3.0 x 10$^9$ |
| Level 2 | 6.0 x 10$^9$ |
| Level 3 | 9.0 x 10$^9$ |
| Level 4 | 12.0 x 10$^9$ |

Dose escalation rules:

| Event at a given dose level | Decision rules |
|---|---|
| No first-course intermediate toxicity or DLT | Enter one patient at the next dose level |

| | |
|---|---|
| First instance of first-course intermediate toxicity | Enter one patient at the next dose level |
| Second instance of first-course intermediate toxicity or first-course DLT | Expand cohort for current dose level and revert to use of 3+3 design for all further cohorts |
| First-course DLT | Expand cohort for current dose level and revert to use of 3+3 design for all further cohorts |

As the expansion rate is variable due to the highly variability of starting material in terms of number and percentages of T cells, in the case of insufficient BET production for the assigned dose level, the patient will be treated at the lower dose or at an intermediate dose level and will be replaced.

Enrollment of patient to the next dose level (according to the table above) will occur after 14 days from infusion of the patient treated in previous cohort.

In the case of reversal to a classic 3+3 design, dose escalation will be done according to the table below:

| **Number of patients with DLT at a given dose level** | **Decision rules** |
|---|---|
| 0 out of 3 | Enter 3 patients at the next dose level |
| 1 out of 3 | Enter 3 more patients at this dose level.<br>- if 0 of these additional 3 patients experience DLT (i.e. 1 out of 6), enter 3 patients at the next dose level<br>- if one or more of these additional 3 patients (i.e., ≥2 out of 6) experience DLT, MTD has been exceeded (MTD = previous dose level) |
| ≥2 out of 3 | MTD has been exceeded (MTD = previous dose level) |

The first two patients in each cohort should be treated with 14 days of delay. In absence of DLT in the first patient during 14 days following BET infusion, the third patient can be enrolled any time. Conversely, in presence of DLT in the first patient, the third patient can be enrolled only after the second patient has completed treatment and no DLT occurred during 14 days following BET infusion. In case a cohort needs to be expanded to more than 3 patients, the additional patients can be enrolled simultaneously. If a patient discontinues the study after BET infusion for reasons other than treatment-related toxicities, an additional patient must be enrolled at the same dose level.

After the identification of the MTD or conclusion of the 4 dose levels in the absence of DLT, an expansion cohort of 9 additional patients will be enrolled. Due to the variability of BET generation among patients, in the expansion cohort patients will receive the actual dose of BET produced expanded from the PBMNC obtained by either PB collected by phlebotomy or by apheresis, according to the review of the dose escalation patients. BET cell dose during dose escalation should not exceed the MTD, if determined. Conversely, if MTD is not reached and the safety profile of BET cell will be consistent with the previous reported toxicity profile of activated T-lymphocytes, in the expansion

| | |
|---|---|
| | phase patients will receive the actual expanded dose of BET |
| DLT/ MTD/OBD | • Dose limiting toxicities (DLT) will be defined as any grade 3 or 4 events occurring within 14 days from BET infusion, that are considered by the investigator to be at least possibly related to therapy. Moderate (or intermediate) toxicity will be defined as grade 2 events that are considered by the investigator to be at least possibly related to therapy<br>• The dose level before the one associated to DLT occurrence is the Maximum Tolerated Dose (MTD).<br>• Optimal biological dose (OBD) is defined as the absolute number of BET cell that will allow a CD3+ count of $\geq$ 600 x$10^6$/L at +90 days after infusion the process of BET cells production (including the starting cell product) resulting in a number of cells in the range of those infused at the highest cohort level of escalation), when the MTD is not reached. This dose will infused in patients enrolled in the extended phase of study. |
| Treatment Duration | Two (i.e. $\geq$48 hours) to five days after the last chemotherapy infusion, BET will be administered.<br>During dose escalation, cells may be frozen in up to 3 bags at the following amounts: 3.0x$10^9$, 3.0x$10^9$, and 6.0x$10^9$ BET cells per bag. The assigned dose will be administered as 1, 2 or 3 bags (max 100 ml).<br>Infusion will be performed at day 0 (1 day of treatment) |
| Sequence of patient entry to dose levels | (see table below) |

| Sequence of patient entry to dose levels (accelerated escalation design) | |
|---|---|
| Pts 1 | Simultaneously |
| Pt 2 | 14 days after treatment of 1ST pt |
| Pt 3 | 14 days after treatment of 2ST pt |
| Pt 4 | 14 days after treatment of 4ST pt |
| **Sequence of patient entry to dose levels (classical 3+3 escalated design)** | |
| Pts 1, 2 | Simultaneously |
| Pt 3 | 14 days after treatment of 1ST pt |
| Pt 4, 5 | 14 days after treatment of 3rd pt |
| Pt 6 | 14 days after treatment of 4rd pt |
| Pt 7, 8 | 14 days after treatment 6th pt |
| Pt 9 | 14 days after treatment of 7rd pt |

| | |
|---|---|
| Pt 10, 11 | 14 days after treatment of $9^{rd}$ pt |
| Pt 12 | 14 days after treatment of $10^{rd}$ pt |

| | |
|---|---|
| Supportive Therapy | The use of rhG-CSF or rhEpo is permitted at the discretion of the Investigator for patient with severe neutropenia or in case of anemia. |
| Safety Assessments | Number, causality and intensity of all adverse events will be evaluated according to the National Cancer Institute (NCI) Common Terminology Criteria for Adverse Events (CTCAE) V4.03 and MedDRA code (current version). |
| Efficacy Assessments | Absolute CD3+ count +90 days after infusion in relation to the absolute number of BET cell infused and its composition (in terms of T-cell subsets and NK cells). OBD of BET will be defined as the absolute number of BET cell that will allow a CD3+ count of $\geq$ 600 x106/L at +90 days after infusion . |
| Sample Size | The study will be conducted according to an accelerated titration dose escalation rule to evaluate the MTD (Simon et al., 1997). Cohorts of one new patient per dose level will be used during the initial accelerated stage of the trial, if the number of BET generated is sufficient. When the first instance of DLT is observed or the second instance of any moderate (grade 2) toxicity, the cohort will be expanded for current dose level and reverted to use of a classic 3+3 design for all further cohorts. After the dose escalation phase, an expansion cohort is planned to further characterize the safety and the immune reconstitution potential of BET cell infusion. The estimated sample size for the expansion cohort is based on the consideration that BET infusion will allow an immune reconstitution ($\geq$600 CD3+ cells x$10^6$/L) after 3 months in at least 50% of the patients, given that without BET support is expected to be recorded in less than 10% in the historical non-treated cohort. With a 1-side test for a proportion, nine evaluable patients will provide 90% power to detect a statistically significant difference with an alpha error of 0.05. With this design, it is expected to enroll 4-24 patients for the dose escalation and 9 patients for the cohort expansion for a total of 13-33 patients. Assuming a drop off rate of less than 5% due to ineligibility for poor clinical response after the third cycle, BET cell contamination by CD19+ cells, and/or inadequate BET cell production for the assigned dose level (during dose escalation) additional 1-2 patients may be enrolled for a total number of up to 35 patients. |
| Inclusion Criteria | *Inclusion criteria to be met at screening for BET production*:<br>1. Male or female patients 18 years or older<br>2. Confirmed diagnosis of the following CD20+ iNHL or CLL according to WHO criteria:<br>o Follicular NHL |

| | |
|---|---|
| | ○ Marginal zone NHL (splenic, extranodal or nodal)<br>○ Lymphocytic lymphoma/CLL without del(17p) or TP53 mutations<br>3. No previous chemotherapy. Previous radiotherapy for localized disease is admitted<br>4. Requirement for treatment:<br>○ For CLL, active disease is defined as meeting at least one of the International Workshop on CLL guidelines (Hallek et al., 2008)<br>○ For iNHL, active disease is defined as meeting at least one of the Groupe d'Etudes des Lymphomes Folliculaires (GELF) criteria (Brice et al., 1997)<br>5. Indication to treatment with either fludarabine, cyclophosphamide and rituximab or bendamustine and rituximab<br>6. Presence of peripheral blood clone ≥10% of total lymphocytes (with absolute lymphocyte count ≥800 x$10^6$/L) at study entrance<br>7. Written informed consent prior to any study procedures being performed<br>*Additional inclusion criteria to be met at study entry (i.e. before BET infusion):*<br>8. Achieving at least a partial response after three chemo-immunotherapy cycles<br>9. Absence of any serious therapy-related complications that might affect interpretation of the results of the study or render the subject at high risk from treatment complications<br>10. Production of adequate BET numbers (counted on CD3+ cells: ≥0.5 x $10^9$)<br>11. For female patients:<br>a) being postmenopausal for at least 1 year before the screening visit, OR<br>b) being surgically sterile, OR<br>c) if they are of childbearing potential, must agree to practice highly effective method of contraception and one additional effective (barrier) method from the time of signing the informed consent until the end of study. Highly effective method of contraception includes: (i) combined (estrogen and progestogen containing) hormonal contraception associated with inhibition of ovulation: oral, intravaginal, transdermal; (ii) progestogen-only hormonal contraception associated with inhibition of ovulation: oral, injectable, implantable (intrauterine device (IUD), intrauterine hormone-releasing system (IUS), bilateral tubal |

| | |
|---|---|
| | occlusion, vasectomized partner, sexual abstinence) OR<br><br>d) must agree to practice true abstinence, when this is in line with the preferred and usual lifestyle of the subject from the time of signing the informed consent until the end of study. [Periodic abstinence (eg, calendar, ovulation, symptothermal, postovulation methods), withdrawal, spermicides only, and lactational amenorrhea are not acceptable methods of contraception. Female and male condoms should not be used together.]<br><br>For male patients, even if surgically sterilized (i.e., status postvasectomy):<br><br>a) with female partners of childbearing potential: must agree to practice barrier contraception (condom with or without spermicide) from the time of signing the informed consent until the end of study and his female partner must agree to practice method of contraception including one of the following: estrogen and progestogen containing hormonal contraception; inhibition of ovulation: oral, intravaginal, transdermal; progestogen-only hormonal contraception associated with inhibition of ovulation: oral, injectable, implantable (intrauterine device (IUD), intrauterine hormone-releasing system (IUS), bilateral tubal occlusion) from the time of signing the informed consent until the end of study.<br><br>b) must agree to practice true abstinence, when this is in line with the preferred and usual lifestyle of the subject from the time of signing the informed consent until the end of study. [Periodic abstinence (eg, calendar, ovulation, symptothermal, postovulation methods), withdrawal, spermicides only, and lactational amenorrhea are not acceptable methods of contraception. Female and male condoms should not be used together.]<br><br>c) must agree to refrain from donating sperm |
| Exclusion criteria | The presence of any of the following will exclude a subject from study enrolment:<br>1. ECOG Performance Status >2<br>2. Active central nervous system (CNS) disease<br>3. Calculated creatinine clearance (by Cockroft-Gault) of < 50 ml/min or serum creatinine > 1.5x ULN<br>4. Concomitant or previous diagnosis of autoimmune hemolytic anemia or thrombocytopenia<br>5. Subjects with active, known or suspected autoimmune disease. Subjects with vitiligo, type |

| | |
|---|---|
| | I diabetes mellitus, residual hypothyroidism due to autoimmune condition requiring the sole hormone replacement are allowed to participate. Psoriasis requiring systemic treatment, or conditions expected to recur at the presence of an external trigger are excluded.<br>6. Known infection with human immunodeficiency virus (HIV) or treponema<br>7. Active hepatitis B virus (HBV) and/or hepatitis C virus (HCV) infections<br>8. Any suspected or known active infection<br>9. History of other diseases, metabolic dysfunctions, physical examination findings, or clinical laboratory findings giving reasonable suspicion of a disease or condition that contraindicates use of an investigational drug or that might affect interpretation of the results of the study or render the subject at high risk from treatment complications<br>10. Residual CD19+ B cells in BET final cell product ≥0.5% |
| Patients replacement | Replacement is accepted in the following cases:<br>✓ If the patient is treated with an dose of cells lower than the assigned dose level (> 10% lower than assigned dose level; intermediate dose between two levels).<br>✓ If a patient discontinues the study during the 90 day interval after BET infusion for reasons other than treatment-related toxicities (DLT/AE), an additional patient must be enrolled at the same dose level. |
| Planned study timelines | Duration of enrolment: 30 months<br>FPI: December 2018<br>Expected LPO: May 2022<br>Expected LPLV: May 2022<br>Duration of whole study (from FPI to LPLV): 46 months |

# 2 STUDY FLOW CHART

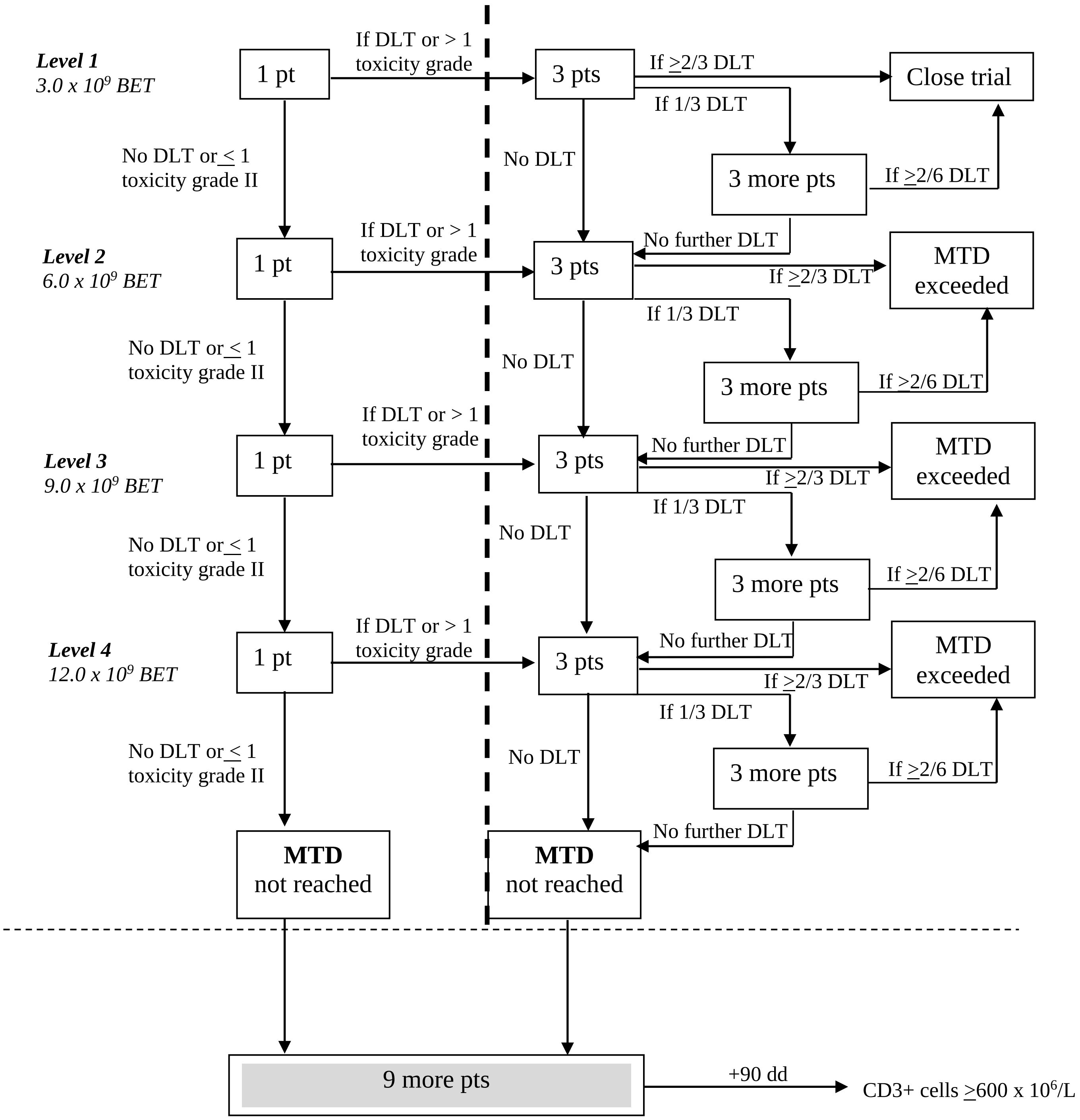

# 3 SCHEDULE OF EVENTS

| | Screening | | Treatment | | | Follow-up |
|---|---|---|---|---|---|---|
| | For BET production (up to 30 days from the first chemotherapy infusion) | For BET infusion (up to 15 days from last planned chemotherapy cycle) | Before BET infusion (up to 8 hours before infusion) | After BET infusion (4 ±1 hours after infusion) | Day +14 after BET infusion (±1 days) | Day +30, Day +90, Day +180 (± 7 days) |
| | **Study Procedures** | | | | | |
| **Informed consent** | X | | | | | |
| **Inclusion/exclusion criteria** | X | X | | | | |
| **Medical history** | X | X | | | | |
| **Physical Examination** | X | X | X | | X | X |
| **Weight** | X | X | | | | |
| **Recording of B-Symptoms** | X | X | | | | |
| **Response Evaluation[1]** | | X | | | | X |
| **Ann Arbor or Rai/Binet stage** | X | | | | | |
| **ECOG performance status[2]** | X | X | X | | X | X |
| **Vital sign[3]** | X | X | X | X | X | X |
| **Adverse events** | | | Continuously assessed | | | |
| **Monitoring of concomitant medications and procedures** | | | Continuously assessed | | | |

| | **Samples/Laboratory Assessments** | | | | | |
|---|---|---|---|---|---|---|
| **Hematology/chemistry**[4] | X | X | X | | X | X |
| **Serologies**[5] | X | | | | | |
| **Pregnancy test**[6] | | X | | | | |
| **HLA typing**[7] | X | | | | | |
| **Blood sample for immunologic parameters**[8] | X | | X | X | | X |
| **12-lead ECG** | X | | X | | | |

1. Response evaluation will be recorded and documented according to disease specific guidelines
2. To be assessed according to Appendix A
3. Vital signs measurement includes blood pressure, heart rate, temperature, respiratory rate, oxygen saturation
4. Laboratory tests includes: complete blood count with differential, calcium, sodium, potassium, magnesium, total and fractionated bilirubin, AST, ALT, alkaline phosphatase, GGT, BUN, creatinine, creatinine clearance (Cockcroft-Gault equation; see Appendix B), acid uric, glucose, amylase, lipase, LDH, serum total protein and electrophoresis, total IgG, IgM and IgA
5. IgG HCV and HIV serology, HBsAg, HBsAb, HBcAb (if HBcAb positive: HBV-DNA), Treponema, CMV IgG and IgM
6. A serum pregnancy test will be performed only for women of childbearing potential at screening
7. HLA typing will be performed after enrollment only if the result of a previous test is not available
8. Approximately 18 mL of whole blood will be collected

* after three chemo-immunotherapy cycles (at least partial response is required for study entry and BET infusion)

# 4 ABBREVIATIONS AND DEFINITIONS OF TERMS

| | |
|---|---|
| AE | Adverse event |
| ATMP | Advanced Therapeutic Medicinal Product |
| BET | Blinatumomab-expanded T cells |
| BR | Bendamustine and rituximab |
| CA | Competent Authority |
| CLL | Chronic lymphocytic leukemia |
| CM | Central memory |
| CMV | Cytomegalovirus |
| CR | Complete response |
| CRO | Contract Research Organization |
| CTCAE | Common terminology criteria for adverse events |
| CTQT | Clinical Trial Quality Team |
| EC | Ethics committee |
| ELISA | Enzyme-linked immunosorbent assay |
| EM | Effector memory |
| IV | Intravenous |
| FCR | Fludarabine, cyclophosphamide and rituximab |
| GCP | Good clinical practice |
| GELF | Groupe d'Etudes des Lymphomes Folliculaires |

| | |
|---|---|
| GMP | Good manufacturing practice |
| HBV | Hepatitis B virus |
| HCV | Hepatitis C virus |
| HLA | Human leukocyte antigen |
| IB | Investigator’s brochure |
| ICF | Informed consent form |
| ICH | International conference on harmonization |
| IL | Interleukin |
| IMP | investigational medicinal product |
| iNHL | Indolent non-Hodgkin lymphomas |
| IRB | Institutional Review Board |
| IWG | International working group |
| LAK | Lymphokine-activated killers |
| MedDRA | Medical dictionary for regulatory activities |
| MTD | Maximum tolerated dose |
| NCI | National Cancer Institute |
| NHL | Non-Hodgkin lymphomas |
| OBD | Optimal biological dose |
| PB | Peripheral blood |
| PBMNC | Peripheral blood mononuclear cells |
| PFS | Progression-free survival |
| PO | Per os |

| | |
|---|---|
| R-CHOP | Rituximab, cyclophosphamide, doxorubicin, vincristine and prednisone |
| R-CVP | Rituximab, cyclophosphamide, vincristine and prednisone |
| SAE | Serious adverse event |
| SCT | Stem-cell transplant |
| SOP | Standard operative procedures |
| TCR | T-cell receptor |
| WHO | World health organization |

# 5 BACKGROUND INFORMATION

## 5.1 Overview of disease pathogenesis, epidemiology and current treatment

Indolent non-Hodgkin lymphomas (iNHL) and chronic lymphocytic leukemia (CLL) are among the most frequent B-cell neoplasms (Ferlay et al., 2013). They include different histologies (i.e. follicular NHL, marginal zone NHL and lymphocytic NHL/CLL) characterized by chronic course and prolonged survival (Swerdlow et al., 2016). While some patients with limited stage disease may be cured, those presenting with advance stage or relapsing after local radiotherapy are generally considered not curable with standard treatments. Early studies have shown that deferring treatment in asymptomatic patients with low tumor burden is not associated to a worse survival and, in many cases, the disease can remain stable for several years (Ardeshna et al., 2003; Brice et al., 1997; CLL Trialists' Collaborative Group, 1999). The usefulness of watchful waiting has been later on confirmed in the Rituximab era (Solal-Celigny et al., 2012). Thus, only patients bearing a high tumor burden disease and/or symptomatic are currently treated with chemo-immunotherapy.

Standard, first line treatment for iNHL, with the exclusion of CLL/lymphocytic lymphoma (LL), includes the use of Rituximab plus chemotherapy. Since no survival advantage has been reported for a specific chemotherapy regimen, several options are currently available and may be chosen according to patient and disease characteristics (Gribben, 2007). Among the most commonly used, bendamustine has shown a remarkable activity with a favorable safety profile. Structurally, bendamustine consists of three moieties: a mechlorethamine group with alkylating properties, a butyric acid side chain that increases water solubility, and a benzimidazole ring that may confer an antimetabolite property (Darwish et al., 2015). Two randomized phase III trial evaluated the association of bendamustine and rituximab (BR) as first line therapy for iNHL in comparison to standard R-CHOP or R-CVP (Flinn et al., 2014; Rummel et al., 2013). The BR regimen showed an overall response rate (ORR) of 93-97% with a complete response (CR) of 28-40%, and a median progression-free survival (PFS) exceeding 4 years. The safety profile of bendamustine/rituximab was distinct from that of R-CHOP/R-CVP, showing less frequently peripheral neuropathy, alopecia, neutropenia and more frequent events of nausea and skin drug-hypersensitivity reactions.

First-line treatment of CLL/LL is currently based on the biologic profile of the disease (Robak et al., 2016). Excluding high risk patients harboring the del(17p) and/or TP53 mutations, first line chemoimmunotherapy options includes the use of either fludarabine, cyclophosphamide and rituximab (FCR) or BR.

Fludarabine, a purine analogue, is prodrug that is converted to the free nucleoside 9-beta-D-arabinosyl-2-fluoroadenine (F-ara-A), which enters cells and accumulates mainly as the 5'-triphosphate, F-ara-ATP (Robak et al., 2006). It acts as antimetabolite by inhibiting DNA polymerase alpha, ribonucleotide reductase and DNA primase, thus inhibiting DNA synthesis. The randomized comparison of FCR and BR regimens has been recently reported (Eichhorst et al., 2016). The ORR were similar for FCR and BR, being respectively of 95% and 96%, however a high proportion of CR was obtained for FCR (40% vs. 31%). The median PFS of FCR arm was 55.2 months, significantly longer of the 41.7 months of the comparator. However, severe neutropenia and infections were more frequently observed with FCR during the study (84% vs. 59%, and 39% vs. 25%, respectively). The increased frequency of infectious complications with FCR was more pronounced in patients older than 65 years. Thus, FCR can be considered the standard regimen for fit patients younger than 65 years, while BR can be considered a valid alterative option.

Despite the good results, treatment with FCR or BR regimens is associated with severe immunosuppression that worsens the immune dysfunctions already present at diagnosis in several patients. In the CLL phase III trial, high frequency of grade 3/4 infections was reported in FCR and BR, being observed in 39% and 25% of the patients, respectively (Eichhorst et al., 2016). In iNHL, infections have been observed in 37-55% of the patients treated with BR, with grade 3/4 events in 7-12% of the cases (Flinn et al., 2014; Rummel et al., 2013).

## 5.2 Introduction to investigational treatment(s) and other study treatment(s)

### *5.2.1 Study drug*

Blinatumomab-expanded T cells (BET) are autologous polyclonal activated T cells expanded in vitro using blinatumomab and rhIL-2. They are an Advanced Therapeutic Medicinal Product (ATMP) for somatic cell therapy in the autologous setting.

The Cell Factory Centro di Terapia Cellulare “G. Lanzani” developed a method to expand and activate *ex vivo* the T lymphocytes present in the peripheral blood from CLL and iNHL patients

for immunotherapeutic purposes. The traditional platform previously used worldwide to expand similar polyclonal T lymphocytes is centered on the concomitant addition of anti-CD3/anti-CD28 antibodies, bound to beads, to activate T cells through their TCR (CD3) and rhIL-2 to promote further T cells proliferation. This method works well when peripheral blood mononuclear cells (PBMC) of healthy donors is used, however, is not appropriate for T cell expansion from samples contaminated with neoplastic cells, because the procedure does not allow destruction of the latter, which remain at the end of culture. Blinatumomab (Blincyto®) is a tandem scFv CD3xCD19 bispecific antibody (BsAb). It binds simultaneously to CD19 on B cells and CD3 on T cells, activate the latter and induce their proliferation (Dreier et al., 2002; Nagorsen and Baeuerle, 2011). Indeed, by several criteria (Ki67 expression, cytokine secretion, cytotoxic granule formation), blinatumomab has comparable potency to anti-CD3/anti-CD28 beads in activating functional T cells and inducing their proliferation in vitro (Wong et al., 2013). In addition, T cells activated with blinatumomab rapidly kill the neoplastic and normal $CD19^+$ B cells present in the culture (Dreier et al., 2002; Loffler et al., 2003). Thus expansion in presence of blinatumomab leads to polyclonal activation and proliferation of T cells and simultaneous elimination of contaminating neoplastic B cells.Therefore, the scientists of Cell Factory ‘G. Lanzani’ have validated such efficient and reproducible expansion of polyclonal peripheral T cells and elimination of contaminating $CD19^+$ B cells by the addition of blinatumomab and rhIL-2 to cultures of PBMCs, without prior T cell enrichment. The expanded population was called BET (Blinatumomab expanded T cells) and the work has been published (Golay et al., 2014). To maximize the expansion potential from relatively small volumes of peripheral blood, based on our previous experience on a similar cell product (Introna et al., manuscript in preparation), we added a small amount of 1% human serum AB to cultures.

The expanded BET final population is composed of CD3+ cells that contain variable proportions of CD4+ and CD8+ cells along with a variable proportion of NK cells. The T-cell receptor (TCR) Vβ families obtained are comparable to the one of the starting population i.e. no restricted clones of T cells are expanded in culture, and includes specificity for common viral antigens such as CMV. Most of the cells are of Th1 functional subtype, with lower frequency of Th2 and very little Th17 and Tregs. Over 95% of BET culture products expressed the α/β, but not γ/δ TCR, indicating that they are standard T cells. Further phenotypic analysis shows that the major

subsets present within both the CD4+ and CD8+ populations were central memory (CM) and effector memory cells (EM), which together represented over 75% of BET at the end of culture. Additionally, BET shows the normalization of the synapse inhibitors CD272 and CD279 molecules, which are usually depressed in CLL patients.

### 5.2.2 Nonclinical experience

Detailed information regarding the nonclinical pharmacology and toxicology of BET can be found in the Investigator's Brochure (IB), version 1, 16/07/2017.

### 5.2.3 Biological activity and pharmacodynamic data in applicable tumor models

Most of the published experiences of clinically tested adoptive transfer of autologous T cells use the anti-CD3/anti-CD28 ex vivo expansion protocol (Garlie et al., 1999; Kalamasz et al., 2004; Levine et al., 1997; Levine et al., 1998; Maus et al., 2002; Rapoport et al., 2005; Rapoport et al., 2009; Stadtmauer et al., 2011). BET cells have never been studied before by any group. Nonetheless, their nature of polyclonally expanded peripheral T cells, lead us to believe that, once *in vivo*, they will behave as described in the traditionally activated anti-CD3/anti-CD28 T lymphocytes and will proliferate, following homeostatic impulse and re-distribute in the lymphoid pool. Thus, we compared the efficiency and overall characteristics of BET expansion method with the anti-CD3/anti-CD28 protocol. Both BET and anti-CD3/anti-CD28 expanded cells contained a variable proportion of $CD4^+$ and $CD8^+$ cells and measurable contamination with NK cells. The anti-CD3/anti-CD28 expanded T cells showed a very similar phenotype to that of BET for most markers analysed. The major phenotypic difference observed was a lower CD27 expression in anti-CD3/anti-CD28 expanded T cells. In conclusion, BET shows strict similarities with the previously clinically tested anti-CD3/anti-CD28 expanded T cells, while it appears greatly superior in the perspective of clinical use in B-cell NHL due to the clearance of contaminating neoplastic B cells.

NK cells present in BET product may only support the control of infections. NK may interact with rituximab administered to patients, and this interaction is expected to be favourable in terms of disease control. In vitro rhIL-2 activated NK cells or PBMCs, also called lymphokine-activated killers (LAK), with or without rituximab, have already been assessed in clinical trials, and limited toxicity was reported for these (Berdeja et al., 2007).

Very few and extremely complicated preclinical models can be used to explore the

possibility to promote immune reconstitution with autologous *ex-vivo* expanded T-cells. Since several clinical experiences with *ex-vivo* expanded T-cells have been previously reported, we indirectly assessed the functionality of our cell product in a mouse B-cell NHL xenograft model (Golay et al., 2014). Upon *in vivo* inoculation, BET retain functional activity: upon engagement with blinatumomab, BET were able to efficiently kill the B-cell NHL cells. BET therefore are able to recognize the tumor target through their TCR (by interaction with anti-CD3 portion of blinatumomab to TCR and CD19 on tumor cells), activate and kill the target tumor. These data therefore indicate that the infused BET maintained their full T cells functionality. Importantly, BET did not show any toxicity in animals, even at high doses and in presence of blinatumomab.

In conclusion, similarly to anti-CD3/anti-CD28 expanded T cells, BET are autologous activated T cells with a mostly central and effector memory phenotype, favorable expression of markers of in vivo persistence (CD27, CD28) and normalized expression of inhibitory receptors (CD279 and CD272). They are polyclonal, contain cells able to recognize common viral antigens such as cytomegalovirus (CMV) and retain the functional activity after infusion *in vivo*. We conclude that BET should have the necessary characteristics to sustain immune-reconstitution in heavily immunocompromised patients and control the infections that frequently affect these patients.

### *5.2.4 Nonclinical risks*

BET cells are autologous cells and therefore are expected to be safe. To the best of our knowledge, no specific studies have ever addressed the toxicity of re-administration of ex vivo expanded autologous polyclonally activated T cells in animal models. More interestingly for the present protocol, no toxicity of BET alone was noted during the course of the experiment in vivo in mice (Golay et al., 2014). Limited toxicity as the occurrence of mild infusion reactions have been reported after infusion of anti-CD3/anti-CD28 expanded autologous T cells (Garlie et al., 1999; Kalamasz et al., 2004; Levine et al., 1997; Levine et al., 1998; Maus et al., 2002; Rapoport et al., 2005; Rapoport et al., 2009; Stadtmauer et al., 2011).

No expected genotoxicity, carcinogenicity, toxicity for reproduction or development is expected nor has ever been reported for in vitro expanded T cells.

### 5.2.5 *Clinical experience*

This is the first in human dose escalation trial of BET infusion in iNHL/CLL patients.

# 6 STUDY RATIONALE

## 6.1 Study rationale and purpose

The use of Rituximab-based combination chemotherapy using drugs with purine analogues moiety (i.e. fludarabine or bendamustine) demonstrated compelling efficacy and is currently a standard treatment of iNHL/CLL patients. Despite the good results, treatment with FCR or BR regimens is associated with severe immunosuppression that worsens the immune dysfunctions already present at diagnosis in several patients. In a large phase III trial evaluating FCR versus BR as first line treatment of CLL, high frequency of severe (grade 3/4) infections was reported in both arms, being observed in 39% and 25% of the patients, respectively (Eichhorst et al., 2016). Additionally, a significant number of severe infections in the FCR group occurred after the end of treatment until 5 months after last chemotherapy. Subjects with iNHL are characterized by a less severe immune dysfunctions compared to CLL patients and, accordingly, less infectious diathesis is reported. Still, infections have been observed in 37-55% of the iNHL patients treated with BR, with grade 3/4 events in 7-12% of the cases (Flinn et al., 2014; Rummel et al., 2013).

While the infectious risk related to neutropenia during treatment can be managed with granulocyte colony stimulating factor (G-CSF) and adequate antibiotic prophylaxis, the immune deficiency related to the severe lymphocyte depletion is more challenging. It is well known that fludarabine and bendamustine treatment results in prolonged lymphocyte depletion, especially in the CD4 subset of T-cells (Darwish et al., 2015; Robak et al., 2006). A grade 3/4 lymphocytopenia has been observed in more than 60-70% of the patients treated with BR, nearly the double in comparison to patients treated with R-CHOP/R-CVP (Flinn et al., 2014; Rummel et al., 2013). The efficient immunological recovery after chemotherapy requires multiple pathways that often undergoes to physiologically age-dependent decline, resulting in a high frequency of long-term immune-deficient patients due to both dysfunctional and/or low absolute T-cell counts (Mackall,

1999). It has been shown that after FCR or BR, patients suffer of a sustained B- and T-cell depletion that can last over the 2-year post treatment period. After FCR, a median time of 24 months is necessary to reach a CD4+ cells recovery of more than 400 cells/µL, while nearly 18 months are necessary to normalize CD8+ cell count and more than 3 years for the Tγδ and NK subsets (Ysebaert et al., 2010). Similar data have been reported after BR treatment (Garcia Munoz et al., 2014; Saito et al., 2015).

Thus, immune reconstitution of iNHL/CLL patients that receive treatment with FCR or BR is a major clinical challenge of adoptive cellular therapy. Restoration of an adequate immune function after first line therapy is today even more important due to the availability of new targeted therapy that can further disrupt normal immunity, further contributing to the risk of opportunistic infections, or of antibodies acting in concert with immune system as checkpoint inhibitors or bispecific antibodies.

It has been previously shown that adoptive transfer of *ex vivo* (anti-CD3/anti-CD28) costimulated autologous T cells can successfully accelerate a robust T-cell recovery early after autologous transplant for multiple myeloma (Rapoport et al., 2005; Rapoport et al., 2009). However, the invariable presence of clonal disease in cell product of iNHL/CLL patients hampered this possibility up to now. We previously reported a simple, GMP-compliant protocol for *ex vivo* expansion of normal T cells from CLL patients peripheral blood for adoptive therapy (Golay et al., 2014). We used the bispecific antibody blinatumomab (anti-CD3/anti-CD19), acting both as T cell stimulator and CLL depletion agent, and human rIL-2. We showed that starting from only 10 mL of peripheral blood, a mean $5.15 \times 10^8$ CD3+ cells can be expanded in 3 weeks with a rapid clearance of CLL contamination. The resulting blinatumomab-expanded T cells (BET) were polyclonal CD4+ and CD8+ T cells and mostly effector and central memory cells. They showed a normalized expression of the synapse inhibitors CD272 and CD279 compared with starting T cells and were functionally active, showing cytotoxicity against CD19+ targets in presence of Blinatumomab *in vitro* and *in vivo*.

On the basis of these data we hypothesize that BET infusion after first-line treatment of iNHL/CLL with either FCR or BR could lead to an adequate immune recovery.

## 6.2 Rationale for the study design

This is a phase I dose escalation, open-label, single center study. The patient population will consist of adults diagnosed with iNHL or CLL in need of first line treatment consisting of either FCR or BR as per investigator assessment. Variable quantity of peripheral blood (PB), that will be around 48ml, will be drawn before first-line treatment and will be used to generate BET under GMP compliant conditions.

Similar cell product (i.e. activated T-lymphocytes) have been previously infused in humans by our group (Introna et al., 2007; Introna et al., 2010) and others (Rapoport et al., 2005; Rapoport et al., 2009), and no significant toxicities have been reported, with the exception of mild infusion reactions. Aiming to limit the proportion of patients treated at subtherapeutic dose levels and reducing trial duration, an accelerated titration dose escalation design will be used to determine the maximum tolerated dose (MTD) of BET over 4 dose levels. After the completion of the dose escalation, an expansion cohort of 9 patients will be treated. Due to the variability of BET generation among patients, in the expansion cohort patients will receive the actual dose of BET expanded, not exceeding the MTD (if determined). Expansion rate is highly variable since starting material derives from a patient population, with different numbers as well as percentages of T and B cells. Similarly, the biological effect on immune reconstitution of BET cells infusion is as well expected to be highly variable. Thus, the dose-response relationship to determine the optimal biological dose (OBD) of BET cells for immune-reconstitution will be evaluated in the whole cohort treated during the expansion phase.

## 6.3 Rationale for dose and regimen selection

Early lymphoid engraftment after autologous stem-cell transplant (SCT) is sustained by the peripheral expansion of the mature, post-thymic T-cells contained into the graft. A median of 17 (±6) $x10^6$ T cells/Kg are usually infused after autologous SCT, and a complete $CD3^+$ cell count is reached after 30 days with a normalized peripheral TCR repertoire (Bomberger et al., 1998). Conversely, it has been shown that a limited diversity of the T-cell repertoire after purged autologous SCT have been observed in patients transplanted with less than 4.0 $x10^3$ T cells/Kg. Restoration of immunity with the use of *in vitro* stimulated autologous T cells have been previously reported (Rapoport et al., 2005; Rapoport et al., 2009). The adoptive transfer of $\leq 5.0x10^9$ of *ex vivo*

(anti-CD3/anti-CD28) costimulated autologous T cells can successfully promote a robust T-cell recovery early after autologous transplant for multiple myeloma without adverse events with the exception of mild infusion reactions.

Based on our large-scale validation experiments, the mean production of $CD3^+$ T cells (BET) starting from 21 ml of whole blood was 3.6 $x10^9$ (range: 0.9-9.4 $x10^9$). Accordingly, in this protocol we will assess the effect of BET infusion on lymphoid immune reconstitution with a starting dose level in the range of the number of the non-stimulated T-cell usually infused during autologous SCT (3.0 $x10^9$), up to the maximum reasonably reachable with the expansion procedure (12.0 x $10^9$). Thus, the dose level of BET cells planned to be assessed in the present study have been calculated basing on both scientific data and the expected production potential of the Cell Factory.

To allow clearance of cytotoxic drugs before BET infusion, study treatment will be infused from 2 ($\geq$48 hours) to 5 days after the last dose of chemotherapy, according to previous experiences in the setting of SCT and cellular therapy (Grupp et al., 2013; Kochenderfer et al., 2012; Long-Boyle et al., 2011).

## 6.4 Rationale for endpoint definition

Restoration of the heterogeneous populations of T cells and the re-establishment of T-cell immunocompetence after cytotoxic antineoplastic therapy is a slow and frequently incomplete process and it has been shown that after FCR or BR, patients suffer of a sustained T-cell depletion that can last over the 2-year post treatment period. Different kinetics of recovery have been observed for the various T-cell subpopulations, and the T-cell count i.e. absolute numbers of CD3+ circulating lymphocytes, can be regarded as key parameters of general T-cell immune reconstitution.

We evaluated the $CD3^+$ counts after FCR or BR treatment in 11 iNHL/CLL patients treated in our Center, finding that a mean of 396 $x10^6$ CD3+ cells/L is reached after 3 to 6 months from the end of treatment (Table 1, unpublished results). Our results are in line with previous reports (Garcia Munoz et al., 2014; Saito et al., 2015; Ysebaert et al., 2010). For the study purpose, we speculate that the increase of $\geq$600 CD3+ cells $x10^6$/L at +90 days in the treated group compared to historical control would be both clinically and statistically significant, given that this end point is reached in

less than 10% of the patients in the historical controls. As a high interpatient variability is expected regarding the effect of BET cell infusion on immune reconstitution, the dose-response relationship, will be determined in the whole cohort and not in every single patient.

Treatment with FCR or BR is associated with increased frequency of infectious complications, in particular in elderly patients, and the frequency and severity of this group of adverse events will be described in the present study. However, since the study is not designed and sized to capture a different trend of infections in treated patients, infectious adverse events will not be included as end points. On the contrary, as immune dysfunctions parameters are closely associated with infectious diathesis, end points of the study will be targeted solely on this aspects.

**Table 1.** Projected $CD3^+$ counts 3 months after FCR or BR

| | ***$CD3^+x10^6/L$*** |
|---|---|
| **Mean** | 396 |
| **Median** | 410 |
| **Standard deviation** | 127 |
| **N** | 11 |

# 7 STUDY OBJECTIVES

## 7.1 Primary Objective

To determine the MTD, and safety profile of BET cells infusion

## 7.2 Secondary Objective(s)

### Secondary safety objectives

To further define the safety and tolerability of BET cells infusion

### Secondary efficacy objectives

- To evaluate the efficiency of BET cells expansion
- To determine the optimal biological dose (OBD) of BET cells
- To characterize the general immune reconstitution after BET infusion
- Evaluation of ex vivo transfer of anti-viral immunity

# 8 STUDY ENDPOINTS

## 8.1 Primary Endpoint

Assessment of Dose Limiting Toxicities (DLTs, defined as any grade 3 or 4 events that are considered by the investigator to be at least possibly related to therapy) observed during 14 days after BET infusion: four escalating dose cohorts will be evaluated and monitored for DLTs (and safety) in order to define MTD.

## 8.2 Secondary Endpoint(s)

### Secondary safety endpoints

Adverse event (AE) and laboratory abnormalities will be continuously assessed throughout the study and reported at each scheduled visit. Description and grading of all adverse events will be based on the NCI –CTCAE v4.03 and MedDra code (current version).

### Secondary efficacy endpoints

- Absolute number of BET expanded and their composition (in terms of T-cell subsets and NK cells) in relation to absolute number of starting CD3+ and CD19+ lymphocytes
- Absolute CD3+ count +90 days after infusion in relation to the absolute number of BET cell infused and its composition (in terms of T-cell subsets and NK cells). OBD of BET will be defined as the absolute number of BET cell that will allow a CD3+ count of $\geq$ 600 x106/L at +90 days after infusion in at least 70% of the patients.
- Absolute numbers of B, T, and NK cells reconstitution at +0 (4 hours), +30, +90 and +180 days after infusion and its correlation with BET cell infused and its composition (in terms of T-cell subsets and NK cells)
- Evaluation of ex vivo transfer of anti-viral immunity in terms of tetramer-based quantification of CMV-specific CD8+ T lymphocytes at +0 (4 hours), +30, +90 and +180

days after infusion (this will be done only for CMV positive patients for whom CMV specific tetramers stain positive in starting peripheral blood or BET).

# 9 STUDY DESIGN

## 9.1 Overview of the study design

This study is a phase I, dose escalation, open-label, single center study, aiming to evaluate the safety and tolerability of BET cells. Secondary efficacy assessment will be performed in terms of achievement of adequate immune reconstitution ($\geq$600 x$10^6$ CD3+ cell/L) at +90 days after infusion in absence of DLTs. Treatment scheme is summarized in Figure 3

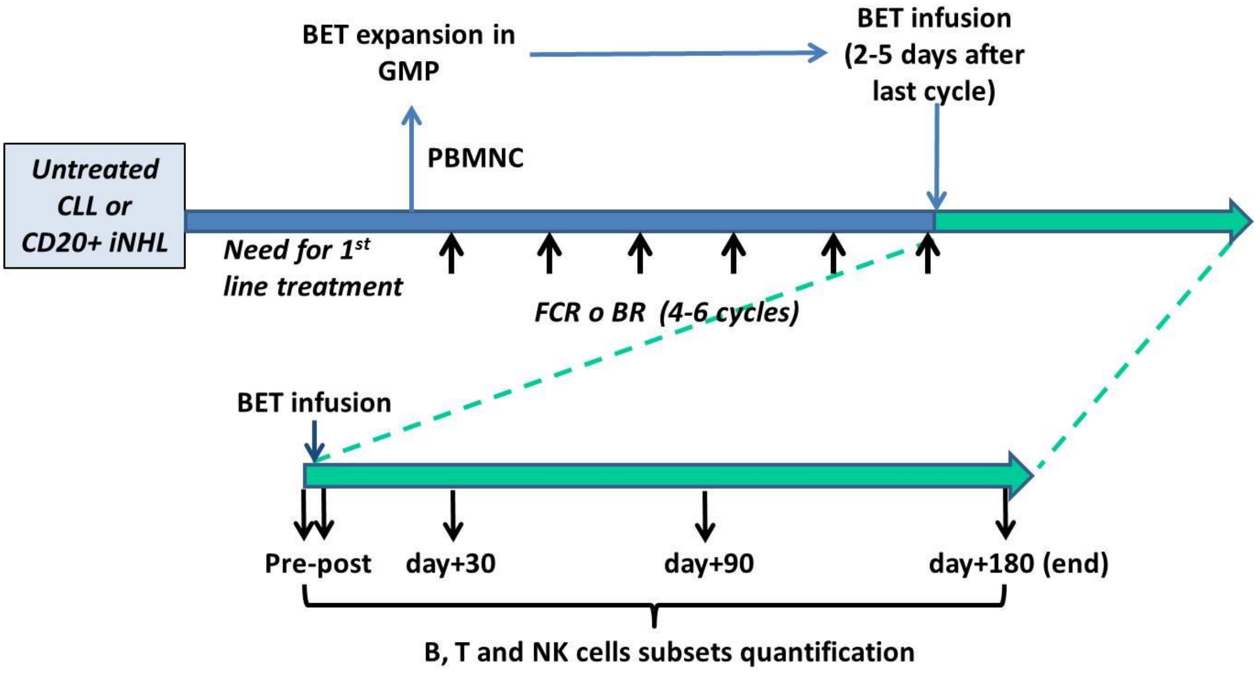


**Figure 3**

The design of the study incorporates a dose escalation phase and a dose expansion. In the dose escalation and expansion phases, patients will be assigned to a dose cohort based on the dose escalation rules as described in the following paragraphs of this section.

Patients will be enrolled in the study after the last planned cycle of chemo-immunotherapy and, if eligibility criteria are met, BET dose level (counted as CD3+ cells) will be assigned during

dose escalation. It is accepted a 10% variability in BET actual dose level. An accelerated titration dose escalation design will be used to assess the MTD of BET cells, if reached. Up to four dose levels will be evaluated until the MTD is reached (Table 3). As the expansion rate is variable due to the highly variability of starting material in terms of number and percentages of T cells, in the case of insufficient BET production for the assigned dose level, the patient will be treated at the lower dose or at an intermediate dose level and will be replaced. In case the number of cells obtained are not sufficient to administer to the patient the starting dose level of 3 x $10^9$, the minimum of 0.5x$10^9$ CD3+ is accepted as the minimum dose for treatment; if it is not reached the patient will be considered as failing screening and excluded from participation to BET trial.

The aggregate data of the dose escalation patients will be analyzed to define the optimal starting material to be used in the expansion cohort. A total of 9 evaluable patients will be treated in the expansion phase to further assess the safety and determine the secondary efficacy endpoints. Due to the variability of BET generation among patients, in the expansion cohort patients will receive the actual dose of BET produced expanded from the PBMNC obtained by either PB collected by phlebotomy or by apheresis, according to the review of the dose escalation patients. BET cell dose during dose explantation should not exceed the MTD, if determined. Conversely, if MTD is not reached and the safety profile of BET cell will be consistent with the previous reported toxicity profile of activated T-lymphocytes, in the expansion phase patients will receive the actual expanded dose of BET.

**Table 3**

| ***Dose level*** | ***BET dose (±10%) (Counted on CD3+ cells)*** |
|---|---|
| 1 (starting dose) | 3.0 x $10^9$ |
| 2 | 6.0 x $10^9$ |
| 3 | 9.0 x $10^9$ |
| 4 | 12.0 x $10^9$ |

Two (i.e. $\geq$48 hours) to five days after the last chemotherapy infusion, BET will be administered.

During dose escalation, cells may be frozen in up to 3 bags at the following amounts: $3.0x10^9$, $3.0x10^9$, and $6.0x10^9$ BET cells per bag. The assigned dose will be administered as 1, 2 or 3 bags (max 100 ml). Immunologic parameters will be evaluated at definite time points i.e. before and 4 hours after the last cell infusion at day 0, at day +30, +90 and +180.

## 9.2 Choice of starting dose and escalated dose

Based on our large-scale validation experiments, the mean production of $CD3^+$ T cells (BET) starting from 21 ml of whole blood was 3.6 $x10^9$ (range: 0.9-9.4 $x10^9$). Accordingly, in this protocol we will assess the effect of BET infusion on lymphoid immune reconstitution with a starting dose level in the range of the number of the non-stimulated T-cell usually infused during autologous SCT (3.0 $x10^9$), up to the maximum reasonably reachable with the expansion procedure (12.0 x $10^9$). Thus, the dose level of BET cells planned to be assessed in the present study have been calculated basing on both scientific data and the expected production potential of the Cell Factory.

## 9.3 Dose escalation rules: sequence and timing of patients inclusion

Patients will be enrolled in the study after the last planned cycle of chemo-immunotherapy and, if eligibility criteria are met, BET dose level will be assigned during dose escalation. Dose escalation during the accelerated part of the trial will be done following the decision rules described below in Table 4. As the expansion rate is variable due to the highly variability of starting material in terms of number and percentages of T cells, in the case of insufficient BET production for the assigned dose level, the patient will be treated at the lower dose or at an intermediate dose level and will be replaced.

**Table 4**

| Event at a given dose level | Decision rules |
| --- | --- |
| No first-course intermediate toxicity or DLT | Enter one patient at the next dose level |
| First instance of first-course intermediate toxicity | Enter one patient at the next dose level |
| Second instance of first-course intermediate toxicity or first-course DLT | Expand cohort for current dose level and revert to use of |

| | |
|---|---|
| | 3+3 design for all further cohorts* |
| First-course DLT | Expand cohort for current dose level and revert to use of 3+3 design for all further cohorts* |

*two patients will be added to complete the cohort level with 3 subject in order to evaluate DLTs according to the classic 3+3 design (see the table 5 below).

Enrollment of patient to the next dose level (according to the table above) will occur after 14 days from infusion of the patient treated in previous cohort.

In the case of reversal to a classic 3+3 design, dose escalation will be done following the decision rules presented in Table 5.

**Table 5**

| Number of patients with DLT at a given dose level | Decision rules |
|---|---|
| 0 out of 3 | Enter 3 patients at the next dose level |
| 1 out of 3 | Enter 3 more patients at this dose level.<br>- if 0 of these additional 3 patients experience DLT (i.e. 1 out of 6), enter 3 patients at the next dose level<br>- if one or more of these additional 3 patients (i.e., ≥2 out of 6) experience DLT, MTD has been exceeded (MTD = previous dose level) |
| ≥2 out of 3 | MTD has been exceeded (MTD = previous dose level) |

The first two patients in each cohort should be treated with at least 14 days of delay. In absence of DLT in the first patient during 14 days following BET infusion, the third patient can be enrolled any time. Conversely, in presence of DLT in the first patient, the third patient can be enrolled only after the second patient has completed treatment and no DLT occurred during 14 days following BET infusion. In case a cohort needs to be expanded to more than 3 patients, the additional patients can be enrolled simultaneously. If a patient discontinues the study after BET infusion for reasons other than treatment-related toxicities, an additional patient must be enrolled at the same dose level.

After the identification of the MTD or conclusion of the 4 dose levels in the absence of DLT, an expansion cohort of 9 additional patients will be enrolled.

## 9.4 Study duration and definition of end of study

The study core includes treatment and follow-up. The screening period will last for each patient approximately 8 months, including up to 2 months before and 6 months of active chemo-immunotherapy treatment. Final eligibility assessment and enrollment will take place at the last chemo-immunotherapy cycle planned. Treatment consist of BET cell infusion at day 0. Study duration will be of 6 months (180 days) starting from BET infusion. For the purpose of this study, the end of the trial is defined as the date of the last visit of the last patient, including follow up.

Based on these consideration thirty (30) months have been foreseen to complete enrollment procedure in the extended phase. It is anticipated that this study will last for approximately 46 months.

## 9.5 Dose-limiting toxicities (DLTs) and intermediate toxicity definitions

Toxicities will be graded according to the NCI CTCAE version 4.03. Dose limiting toxicities (DLT) will be defined as any grade 3 or 4 events that are considered by the investigator to be at least possibly related to therapy. Moderate toxicity will be defined as grade 2 events that are considered by the investigator to be at least possibly related to therapy.

Although DLT-like events may occur at any point during treatment, only DLTs defined during the 14-days after BET infusion will influence decisions regarding dose escalation or expansion of a dose level. Patients will be monitored through the study for treatment-related toxicities. A more conservative dose escalation with evaluation of intermediate dose levels, expansion of an existing dose level, and alternative regimens/schedule are all permissible following discussions between the Sponsor and the Investigators, if such measures are needed for patient safety or for a better understanding of the dose-toxicity and dose-exposure relationship of BET.

## 9.6 Escalation schema

According to the rules described above, the escalation schema could be presented with the following table

| Dose level | BET dose (Counted on CD3+ cells) | N. of patients (accelerated escalation) | N. of patients For Reversal to 3+3 classic design | N. of patients (3+3 classic design) |
|---|---|---|---|---|
| 1 (starting dose) | $3.0 \times 10^9$ | 1 | +2 | 3 (+3) |
| 2 | $6.0 \times 10^9$ | 1 | +2 | 3 (+3) |
| 3 | $9.0 \times 10^9$ | 1 | +2 | 3 (+3) |
| 4 | $12.0 \times 10^9$ | 1 | +2 | 3 (+3) |

## 9.7 Sequence of patients enrolled

At each dose level, patients will enter according to the following sequence:

Accelerated escalation design:

| Sequence of patient entry to dose levels | |
|---|---|
| Pts 1 | Simultaneously |
| Pt 2 | 14 days after treatment of 1ST pt |
| Pt 3 | 14 days after treatment of 2ST pt |
| Pt 4 | 14 days after treatment of 4ST pt |

Classical 3+3 escalated design:

| Sequence of patient entry to dose levels | |
|---|---|
| Pts 1, 2 | Simultaneously |
| Pt 3 | 14 days after treatment of 1ST pt |
| Pt 4, 5 | 14 days after treatment of 3rd pt |
| Pt 6 | 14 days after treatment of 4rd pt |
| Pt 7, 8 | 14 days after treatment 6th pt |
| Pt 9 | 14 days after treatment of 7rd pt |
| Pt 10, 11 | 14 days after treatment of 9rd pt |
| Pt 12 | 14 days after treatment of 10rd pt |

# 10 STUDY POPULATION

## 10.1 Subject Selection

The patient population will consist of adults diagnosed with iNHL or CLL in need of first line treatment consisting of either FCR or BR as per investigator assessment. Variable quantity of PB, around 48ml, will be drawn by phlebotomy or apheresis before first-line treatment and will be used to generate BET under GMP compliant conditions. Cell product will be evaluated for the presence of NHL clones (CD19+) by flow cytometry, patients with measurable (≥0.5%) residual B-cells will be excluded from the study.

### 10.1.1 Subject Inclusion Criteria

Subjects must meet all the following inclusion criteria to be eligible for enrolment into the study:

<u>Inclusion to be met at screening for BET production:</u>

1. Male or female patients 18 years or older
2. Confirmed diagnosis of the following CD20+ iNHL or CLL according to WHO criteria:
    - Follicular NHL
    - Marginal zone NHL (splenic, extranodal or nodal)
    - Lymphocytic lymphoma/CLL without del(17p) or TP53 mutations
3. No previous chemotherapy. Previous radiotherapy for localized disease is admitted
4. Requirement for treatment:
    - For CLL, active disease is defined as meeting at least one of the International Workshop on CLL guidelines (Hallek et al., 2008)
    - For iNHL, active disease is defined as meeting at least one of the Groupe d'Etudes des Lymphomes Folliculaires (GELF) criteria (Brice et al., 1997)
5. Indication to treatment with either fludarabine, cyclophosphamide and rituximab or bendamustine and rituximab
6. Presence of peripheral blood clone ≥10% of total lymphocytes (with absolute lymphocyte count ≥800 x$10^6$/L) at study entrance
7. Written informed consent prior to any study procedures being performed

Additional inclusion criteria to be met at study entry (i.e. before BET infusion):

8. Achieving at least a partial response after three chemo-immunotherapy cycles
9. Absence of any serious therapy-related complications that might affect interpretation of the results of the study or render the subject at high risk from treatment complications
10. Production of adequate BET numbers (counted on CD3+ cells: $\geq$0.5 x $10^9$)
11. For female patients:

    a) being postmenopausal for at least 1 year before the screening visit, OR

    b) being surgically sterile, OR

    c) if they are of childbearing potential, must agree to practice highly effective method of contraception and one additional effective (barrier) method from the time of signing the informed consent until the end of study. Highly effective method of contraception includes: (i) combined (estrogen and progestogen containing) hormonal contraception associated with inhibition of ovulation: oral, intravaginal, transdermal; (ii) progestogen-only hormonal contraception associated with inhibition of ovulation: oral, injectable, implantable (intrauterine device (IUD), intrauterine hormone-releasing system (IUS), bilateral tubal occlusion, vasectomised partner, sexual abstinence) OR

    d) must agree to practice true abstinence, when this is in line with the preferred and usual lifestyle of the subject from the time of signing the informed consent until the end of study. [Periodic abstinence (eg, calendar, ovulation, symptothermal, postovulation methods), withdrawal, spermicides only, and lactational amenorrhea are not acceptable methods of contraception. Female and male condoms should not be used together.]

    For male patients, even if surgically sterilized (i.e., status postvasectomy):

    a) with female partners of childbearing potential: must agree to practice barrier contraception (condom with or without spermicide) from the time of signing the informed consent until the end of study and his female partner must agree to practice method of contraception including one of the following: estrogen and progestogen containing hormonal contraception; inhibition of ovulation: oral, intravaginal, transdermal; progestogen-only hormonal contraception associated with inhibition of ovulation: oral,

injectable, implantable (intrauterine device (IUD), intrauterine hormone-releasing system (IUS), bilateral tubal occlusion) from the time of signing the informed consent until the end of study.

b) must agree to practice true abstinence, when this is in line with the preferred and usual lifestyle of the subject from the time of signing the informed consent until the end of study. [Periodic abstinence (eg, calendar, ovulation, symptothermal, postovulation methods), withdrawal, spermicides only, and lactational amenorrhea are not acceptable methods of contraception. Female and male condoms should not be used together.]

c) must agree to refrain from donating sperm

### 10.1.2 Subject Exclusion Criteria

The presence of any following will exclude a subject from study enrolment:

1. ECOG Performance Status >2.
2. Active central nervous system (CNS) disease.
3. Calculated creatinine clearance (by Cockroft-Gault) of < 50 ml/min or serum creatinine > 1.5x ULN
4. Concomitant or previous diagnosis of autoimmune hemolytic anemia or thrombocytopenia
5. Subjects with active, known or suspected autoimmune disease. Subjects with vitiligo, type I diabetes mellitus, residual hypothyroidism due to autoimmune condition requiring the sole hormone replacement are allowed to participate. Psoriasis requiring systemic treatment, or conditions expected to recur at the presence of an external trigger are excluded.
6. Known infection with human immunodeficiency virus (HIV) or treponema
7. Active hepatitis B virus (HBV) and/or hepatitis C virus (HCV) infections
8. Any suspected or known active infection
9. History of other diseases, metabolic dysfunctions, physical examination findings, or clinical laboratory findings giving reasonable suspicion of a disease or condition that contraindicates use of an investigational drug or that might affect interpretation of the results of the study or render the subject at high risk from treatment complications
10. Residual CD19+ B cells in BET final cell product ≥0.5%

## 10.2 Screening failures

For all screened patients, a “Subject Screening Log” will be completed and in case of screening failure the reason(s) for failure will be documented CRFs have to be completed only for registered patients.

Screening failure occurs when one or more of inclusion/exclusion criteria are not satisfied. Particularly, regarding the inclusion criteria concerning the production of cells a screening failure occurs when cells production does not allow to administer at least the minimum dose of 0.5 x $10^9$ $CD3^+$. In all other cases when assigned dose level is not reached, the patients will be included in the study and treated with the actual number of cells obtained.

## 10.3 Replacements

Replacements occur when:

- ✓ Patients are treated with an dose of cells lower than the assigned dose level (> 10% lower than assigned dose level; intermediate dose between two levels).
- ✓ Patients are withdrawn from treatment during the 90 days interval after BET infusion for reasons other than DLT/intermediate AE occurrence.

# 11 ENROLLMENT PROCEDURES

Before registration at study entry all patients who are potential candidate to be enrolled must have given a written informed consent for the study. The following logs must be maintained at study site and kept in the Investigator File: a “Patient Screening and enrollment Log”, to document the identification of subjects who enter screening and to document chronological enrolment and a “Patient Identification Code List” for all subjects registered to maintain the correlation with the patient's full identification data (name, surname - confidential). Before registration in the study, all the pre-treatment evaluations (i.e. assessment of response after chemotherapy) must be reported on the eCRF "Screening Form”. Upon review of all inclusion/exclusion criteria, if the patient is eligible for BET expansion, a progressive “Registration Number” and dose level, is centrally assigned by the electronic system provided by the Sponsor of the study.

Any controversial eligibility assessment will be discussed with the Principal Investigator and the Project Manager.

# 12 STUDY TREATMENT

## 12.1 Trial Product

The investigational medicinal products (IMP) for this study are autologous polyclonal activated T cells expanded in vitro using blinatumomab and rhIL-2, named BET. The IMP will be supplied in one or more cryopreserved sterile bags.

The IMP is frozen in heat-sealed ethyl vinyl acetate double freezing bags (CryoMACS Freezing Bags from Milteny), designed for cryopreservation of haematopoietic progenitor cells. CryoMACS freezing bags are CE-marked medical devices. The cells containing inner bag is sealed by heat using a tube sealer. The inner bag is then inserted in an outer bag, which is then sealed using a bag sealer; both sealings are verified mechanically. The excipients are clinical grade DMSO (10%) and human homologous plasma. The final volume is up to 50 ml. Bags are labeled immediately after production. Labels are produced according to need, so no unused labels remain after the procedure, and include the following information: identification of the production site, identification of the product, lot number, number of total BET (counted as CD3+ cells) in each bag, name and surname and date of birth of recipient, date of freezing, date of expiry, excipients, information on storage condition and on the methods of administration. The cryopreserved bags are stored at the Laboratory of Cellular Therapy "*G. Lanzani*" in liquid nitrogen tanks constantly monitored for temperature and nitrogen levels and placed in a dedicated room with limited and controlled access, until the bag is supplied for infusion.

## 12.2 Drug preparation (if not included in a specific manual)

After written informed consent and documentation of eligibility criteria, PBMNC will be obtained by either PB collected by phlebotomy or by apheresis. BET will be prepared and processed according to GMP procedure in the AIFA approved Cell Factory i.e. Laboratory of Cellular Therapy "*G. Lanzani*" located at "*ASST-Papa Giovanni XXIII*" of Bergamo (authorization n° aM-

57/2016, 19-Apr-2016).

The Investigator or designee will complete the bag request form. An operator of the Laboratory of Cell Therapy "*G. Lanzani*" will verify the availability of the requested cells and, only after this confirmation, the patient could be enrolled. The day of BET administration, the operator at Laboratory of Cell Therapy "*G. Lanzani*" will prepare the documentation, including a copy of the signed Certificate of Analysis, take the bag from the container, put it immediately in the validated shipment cryocontainer, activate the temperature data logger according to validated standard operative procedures (SOPs), and will inform the dedicated transport service. Control of the bag (identity and integrity of bag and labels) will be performed also by a second operator.

The transporter will sign the shipment form for acceptance and will immediately deliver the bag to the study site. Upon arrival, the personnel in the wards will control the integrity and identity of the cell products as well as their proper labeling, the correct temperature during transport, as detailed in specific SOPs, and will proceed to the ward for the bag thawing and infusion according to written SOPs. All procedures for transport, thawing, infusion, adverse events and their communication to the producing Laboratory, quality testing (viability) of the thawed product, measures to be taken in case on impossibility to perform the infusion within 4 hours of receipt are carried out according to written SOPs.

## 12.3 Treatment Dose and Schedule

It is accepted a 10% variability in BET actual dose level. An accelerated titration dose escalation design will be used to assess the MTD of BET cells, if reached. Up to four dose levels will be evaluated until the MTD is reached (Table 3). As the expansion rate is variable due to the highly variability of starting material in terms of number and percentages of T cells, in the case of insufficient BET production for the assigned dose level, the patient will be treated at the lower dose or at an intermediate dose level and will be replaced. The aggregate data of the dose escalation patients will be analyzed to define the optimal starting material to be used in the expansion cohort. A total of 9 evaluable patients will be treated in the expansion phase to further assess the safety and determine the secondary efficacy endpoints. Due to the variability of BET generation among patients, in the expansion cohort patients will receive the actual dose of BET produced expanded

from the PBMNC obtained by either PB collected by phlebotomy or by apheresis, according to the review of the dose escalation patients. BET cell dose during dose expansion should not exceed the MTD, if determined. Conversely, if MTD is not reached and the safety profile of BET cell will be consistent with the previous reported toxicity profile of activated T-lymphocytes, in the expansion phase patients will receive the actual expanded dose of BET.

**Table 3**

| *Dose level* | *BET dose* (Counted on CD3+ cells)* |
|---|---|
| 1 (starting dose) | $3.0 \times 10^9$ |
| 2 | $6.0 \times 10^9$ |
| 3 | $9.0 \times 10^9$ |
| 4 | $12.0 \times 10^9$ |

Two (i.e. $\geq$48 hours) to five days after the last chemotherapy infusion, BET will be administered. During dose escalation, cells may be frozen in up to 3 bags at the following amounts: $3.0 \text{x} 10^9$, $3.0 \text{x} 10^9$, and $6.0 \text{x} 10^9$ BET cells per bag. The assigned dose will be administered as 1, 2 or 3 bags (max 100 ml).

## 12.4 Duration of Treatment and Administration Management

The infusion will be performed in one day and will last for 30-180 minutes. The Investigator or designee must thaw, infuse the cells and monitor the patient throughout the procedure. Administration of the study drug will be documented and, in case of adverse events (AEs) during infusion leading to treatment discontinuation, the amount dispensed to subjects will be reported and the bag returned to the producing Laboratory where it will be destroyed. The necessary equipment and emergency drugs (hydrocortisone, chlorpheniramine, adrenaline) must be available. The bags will be thawed immediately before use and the contents administered as rapidly as possible after

thawing. Viability and recovery after thawing will be assessed. It is the responsibility of the Investigator to ascertain that the aliquots are correctly labeled, and that the labeling corresponds to the Cell Therapy Laboratory documentation. Chlorphenamine (10 mg ev) will be given before infusion along with acetaminophen (1 gr po). The use of steroids in this situation should be avoided but it is admitted in case of reaction, if judged clinically mandatory by the Investigator. Salbutamol nebulizers or oxygen should be given if dyspnea or cyanosis occurs. The Investigator will document and report any adverse event taking place after cell therapy product infusion.

## 12.5 Drug Accountability

IMP will be provided the Laboratory of Cell Therapy “G. Lanzani”. The study site will acknowledge receipt of IMP to confirm the shipment condition and content. The Investigator or designee is responsible for the thawing and infusion of IMP. In case of AEs leading to treatment interruption, unused cell product will either be disposed of at the study site according to the study site’s institutional standard operating procedure or returned to the Laboratory of Cell Therapy “G. Lanzani” with the appropriate documentation. Accurate records of all IMP received at, dispensed from, returned to, and disposed of by the study site should be recorded

## 12.6 Treatment Dose Modifications

No dose reduction is allowed in this study.

## 12.7 Concomitant Medications and Other Therapy

The following medications and procedures are prohibited or limited during the study:

- Treatment with any antineoplastic chemotherapy or investigational agents
- Radiation therapy (note that, in general, the requirement for local radiation therapy indicates disease progression).
- Treatment with any immunosuppressive drug.

- Treatment with high dose corticosteroids within 5 days before or after the BET infusion. Daily dose equivalent to 10 mg oral prednisone is permitted. Corticosteroids for topical use or in nasal spray or inhalers are allowed.

The use of rhG-CSF or rhEpo is permitted at the discretion of the Investigator for patient with severe neutropenia or in case of anemia.

# 13 SUBJECT WITHDRAWAL FROM STUDY PARTICIPATION

In accordance with the current revision of the Declaration of Helsinki and other applicable regulations, a subject (or a legally acceptable representative) has the right to withdraw from the study at any time and for any reason without prejudice to his or her future medical care by the physician or at the institution.

Discontinuation will be recorded at the end of treatment. Reasons that a patient may discontinue participation in this clinical study may be considered to constitute one of the following items:

- Adverse event
- Progressive disease
- Symptomatic deterioration (at Investigator's discretion)
- Unsatisfactory therapeutic response
- Study terminated by Sponsor
- Withdrawal by subject
- Lost to follow-up
- Other

The primary reason for study discontinuation will be recorded on the eCRF: at the time of withdrawal, the investigator should schedule the End of Treatment and Follow-up visits in agreement with the patient.

# 14 TREATMENT ASSESSMENT

Patients will be evaluated at scheduled visits over the following study periods: Screening, Treatment, and Follow-up. Evaluations during the Screening period are divided into two parts, the eligibility for BET production, to be conducted within 30 days before administration of the first dose of standard chemotherapy, and final eligibility to the study (i.e. BET infusion), to be conducted within 15 days before the last planned chemo-immunotherapy cycle. Tests and procedures should be performed in a time window of 7 days for all scheduled visits, unless otherwise specified. Refer to the Schedules of Events for timing of assessments. Additional details are provided as necessary in the sections that follow.

## 14.1 Pre-Treatment Evaluations

***Informed consent***

Each patient must provide written informed consent before any study-required procedures are conducted, unless those procedures are performed as part of the patient’s standard care.

***Screening***

Screening period is divided into two parts: assessment of patient eligibility for BET production, conducted within 30 days before administration of the first dose of standard chemotherapy, and final eligibility to the study (i.e. BET infusion), within 15 days before the last planned chemo-immunotherapy cycle.

PBMNC blood will be obtained for BET production only in patient who met all the inclusion and exclusion criteria (except BET production criteria). Patients will be treated according to local practice with either FCR or BR chemo-immunotherapy for at least 4 cycles and no more than 6 cycles. At the last planned cycle, final review of inclusion and exclusion criteria will be assessed (including BET production criteria), eligible patients will be enrolled, and dose level assigned.

The following exam must be performed according to scheduled time of table reported in Section 3:

- Medical history
- Response evaluation will be recorded and documented according to disease specific guidelines
- ECOG (Appendix A)
- Physical examination
- Vital signs including: blood pressure, heart rate, temperature, respiratory rate, oxygen saturation
- Weight
- B-Symptoms
- Ann arbor stage
- 12-lead ECG
- Serologies: IgG HCV and HIV serology, HBsAg, HBsAb, HBcAb (if HBcAb positive: HBV-DNA), Treponema, CMV IgG and IgM
- Laboratory tests including: complete blood count with differential, calcium, sodium, potassium, magnesium, total and fractionated bilirubin, AST, ALT, alkaline phosphatase, GGT, BUN, creatinine, creatinine clearance (Cockcroft-Gault equation; see Appendix B), acid uric, glucose, amylase, lipase, LDH, serum total protein and electrophoresis, total IgG, IgM and IgA
- Serum pregnancy test will be performed for women of childbearing potential at screening up to 15 days from last planned chemotherapy (before BET infusion)
- HLA typing will be performed after enrollment only if the result of a previous test is not available
- Immunologic parameters: absolute B, T (CD4+/CD8+) and NK cell counts; CMV-specific CD8+ T lymphocytes

## 14.2 On Study Evaluations

The following exam must be performed up to 8 hours before BET infusion according to scheduled time of table reported in Section 3:

:

- ECOG (Appendix A)
- Physical examination
- Vital signs including: blood pressure, heart rate, temperature, respiratory rate, oxygen saturation
- Laboratory tests including: complete blood count with differential, calcium, sodium, potassium, magnesium, total and fractionated bilirubin, AST, ALT, alkaline phosphatase, GGT, BUN, creatinine, creatinine clearance (Cockcroft-Gault equation; see Appendix B), acid uric, glucose, amylase, lipase, LDH, serum total protein and electrophoresis, total IgG, IgM and IgA
- Standard HLA typing will be performed after enrollment only if the result of a previous test is not available
- 12-lead ECG
- Immunologic parameters: absolute B, T (CD4+/CD8+) and NK cell counts; CMV-specific CD8+ T lymphocytes
- Adverse events are monitored any time

The following exam must be performed 4(±1) hours after BET infusion according to scheduled time of table reported in Section 3:

- Vital signs including: blood pressure, heart rate, temperature, respiratory rate, oxygen saturation

- Immunologic parameters: absolute B, T (CD4+/CD8+) and NK cell counts; CMV-specific CD8+ T lymphocytes

The following exam must be performed 14 days after BET infusion according to scheduled time of table reported in Section 3:

- ECOG (Appendix A)
- Physical examination
- Vital signs including: blood pressure, heart rate, temperature, respiratory rate, oxygen saturation
- Laboratory tests including: complete blood count with differential, calcium, sodium, potassium, magnesium, total and fractionated bilirubin, AST, ALT, alkaline phosphatase, GGT, BUN, creatinine, creatinine clearance (Cockcroft-Gault equation; see Appendix B), acid uric, glucose, amylase, lipase, LDH, serum total protein and electrophoresis, total IgG, IgM and IgA
- Adverse events are monitored any time

## 14.4 Follow-Up Evaluations

The following exam must be performed after BET infusion and follow-up according to scheduled time of table reported in Section 3:

- Physical examination
- Response evaluation will be recorded and documented according to disease specific guidelines
- ECOG (Appendix A)
- Vital signs including: blood pressure, heart rate, temperature, respiratory rate, oxygen saturation

- Laboratory tests including: complete blood count with differential, calcium, sodium, potassium, magnesium, total and fractionated bilirubin, AST, ALT, alkaline phosphatase, GGT, BUN, creatinine, creatinine clearance (Cockcroft-Gault equation; see Appendix B), acid uric, glucose, amylase, lipase, LDH, serum total protein and electrophoresis, total IgG, IgM and IgA
- Immunologic parameters: absolute B, T (CD4+/CD8+) and NK cell counts; CMV-specific CD8+ T lymphocytes
- Adverse events are monitored any time

## 14.5 Details of Individual Assessments

### *14.5.1 Safety endpoints assessment*

Primary objective of the study is to determine the safety profile and tolerability of BET cell infusion. A visit is scheduled at the end of the 14-days DLT period to evaluate the occurrence of treatment-related toxicities (see Schedule of events). Adverse event (AE) and laboratory abnormalities will be continuously assessed throughout the study (see Section 11 and Schedule of events).

### *14.5.2 Efficacy endpoints assessment*

Secondary objectives of the study are to evaluate patient immunological reconstitution and BET cell production process. Approximately 18 mL of whole blood will be drawn by phlebotomy for determination of the immune parameters, as detailed in the Schedules of Events. Parameters under evaluation include:

- CD3+/CD4+, CD3+/CD8+, CD19+ and CD56+ lymphocyte absolute count by flow cytometry
- Tetramer-based quantification of CMV-specific CD8+ T lymphocytes by flow cytometry (in eligible patients)

### *14.5.3 Other clinical evaluation, laboratory tests and follow-up*

This study will not impose any additional burden on the patients or the hospital service in

terms of clinical evaluation or laboratory tests comparing to all patient in need of chemo-immunotherapy treatment for iNHL/CLL. The study endpoints will be formally collected in appropriate eCRFs.

#### *14.5.4 Disease response assessment guidelines*

The clinical response after the third chemo-immunotherapy cycle and after last cycle will be documented in the appropriate eCRF. Definition of response will be based according to standard of care disease-specific response criteria, as follows:

- Definition of response in malignant lymphomas: according to the Lugano 2014 criteria (Cheson et al., 2014)
- Definition of response in chronic lymphocytic leukemia: according to IWG 2008 (Hallek et al., 2008)

# 15 SAFETY ASSESSMENTS

The toxicities will be evaluated according to the National Cancer Institute (NCI) Common Toxicity Criteria (CTCAE) V4.03 and MedDRA code (current version). All baseline tumor-related signs and symptoms assessment are to be performed prior to the start therapy and will be followed at each visit. Although a measure of efficacy, these will be reported on the Baseline Signs and Symptoms CRF pages and on the Adverse Events pages in case of symptoms worsening if appropriate. The CTCAE Grade must be recorded at each visit (while present) even if the event has improved or remains stable.

## 15.1 Pre-existing condition

Any new condition and any worsening of pre-existing condition occurring after Informed Consent signature and before study treatment start are to be considered for Serious Adverse Event (SAE) reporting (see 15.4).

## 15.2 Adverse Event Assessment

Adverse Events (AE)

An adverse event is any untoward medical occurrence in a patient that is administered a drug or biologic (medical product); the event does not necessarily have a causal relationship with that treatment or usage. Adverse events include the following:

● All suspected medication adverse reactions

● All reaction from medication overdose, abuse, withdrawal, sensitivity, or toxicity

● Apparently unrelated illness, including the worsening of a pre-existing illness

● Injury or accidents. Note that if a medical condition is known to have caused an injury or accident (e.g., a fracture due to a fall secondary to dizziness), the medical condition (dizziness) and the injury (fracture) should be reported as 2 separate adverse events.

● Laboratory abnormalities and abnormalities in physiological testing or physical examination findings that require clinical intervention (e.g. therapeutic measures, IMP dose and/or schedule changes) or further investigation (beyond ordering a repeat [confirmatory] test) or that are considered clinically significant by the investigator

Any events

Each adverse event is to be classified by the investigator as serious or non-serious. This classification of the gravity of the event determines the reporting procedures to be followed. Progression of disease intended as increase of tumor burden should not be reported as an adverse event, while any clinical sign/symptom/illness associated with malignant disease progression should be recorded as adverse event.

Serious Adverse Events (SAE)

The definition of seriousness is based on the patients/event outcome or action criteria associated with events that pose a threat to patient's life or functioning.

An adverse event that meets one or more of the following definition is classified as serious:

● Results in death

● Is life-threatening (i.e., the patient was at risk of death at the time of the event; it is not referred to cases in which the event might have caused death if it was more severe)

● Requires in-patient hospitalization or prolongation of existing hospitalization

● Results in persistent or significant disability/incapacity

- Is a congenital anomaly/birth defect

Important adverse events that may not result in death, may not be life-threatening, or do not require hospitalization may be considered serious when, based on the investigator's medical judgment, they may jeopardize the patient or require medical or surgical intervention to prevent one of the outcomes listed above.

Events not considered serious include hospitalizations which are:

a) elective and planned before entry into the study

b) emergency and do not result in overnight hospitalization, unless fulfilling the criteria above

c) for the routine treatment of study indication and not associated with any deterioration in condition.

In all other cases, the hospitalization seriousness criterion is met as long as hospitalization is required which does not necessarily correspond to the actual hospitalization period (e.g., a patient might be admitted to hospital two days after this measure was required because the investigator was not informed of the patient's conditions)

AEs that must be considered serious by default regardless if they meet the seriousness criteria.

## 15.3 Adverse Event Reporting Period

All AEs will be assessed at each visit and will be recorded by the Investigator from the time of informed consent signature until 30 days after the IMP infusion for patients withdrawing the trial; the other patients will be monitored for safety until their last visit.

All AEs will be recorded on the relevant form of the eCRF and in the patient's source documents.

AEs assessed by the investigators as unrelated to the IMP must be followed until resolution or until 30 days after the last IMP dose whichever occurs earlier. AEs assessed by the Investigator as related to the IMP and SAEs of any causality must be followed until resolution or death if this occurs beyond the AE reporting period defined above, unless the patient is lost to follow-up or start a new systemic anti-tumor therapy or the event has stabilized and is assessed as chronic by the Investigator.

## 15.4 Reporting procedures for Adverse Event

Each adverse event is to be classified by the investigator as SERIOUS or NON-SERIOUS. This classification of the gravity of the event determines the reporting procedures to be followed. If a serious adverse event occurs, the is to be notified, using the SAE report form and completing all sections concerning seriousness, within 24 hours of awareness of the event by the investigator. If the initial report is incomplete or the event is still ongoing at the time of reporting or if new significant information becomes available, this report is to be followed by submission of follow-up information within 5 calendar days after the initial notification. Reporting requirements for adverse events are summarized in the following table.

**REPORTING REQUIREMENTS FOR ADVERSE EVENT**

| Gravity | Reporting Time | Type of Report |
|---|---|---|
| SERIOUS | Within 24 hours from awareness by the investigator | e-case report form SAE + SAE report form (fax) |
| | Within 5 calendar days from initial report | e-CRF SAE Follow-up o + Follow-up SAE report form (fax) |
| NON SERIOUS | Per case report form submission procedure | e-CRF AE |

All SAEs report form must be sent by the investigators to the following address of Safety Desk of ASST-Papa Giovanni XXIII: mlorini@asst-pg23.it .

The personnel designated at Safety Desk is responsible to assess each SAE reported by the study Investigators to identify any suspected unexpected serious adverse reactions (SUSAR), i.e. serious adverse events considered at least possible associated to the study treatment by either the Investigator or the Chair and not mentioned in the IMP reference document: Investigator's Brochure current updated Version.

Safety Desk of ASST-Papa Giovanni XXIII will issue a SUSAR (Suspect Unexpected Serious Adverse Reaction) notification whenever appropriate, and submit it to the supplier product. as well as to Eudravigilance, the concerned Ethics Committees and Competent Authorities.

Follow-up information is to be reported on a new eCRF form. A follow-up report is to be filled in, not only to complete the information provided on the initial report but also to modify any incorrect data.

## 15.5 Recording Adverse Events in the Case Report Forms

AEs can be directly assessed by the Investigator during a clinical visit or based on laboratory/Instrumental examinations..

- Pre-existing Conditions

A pre-existing condition (i.e., a disorder starting before the adverse event reporting period) should not be reported as an adverse event unless the condition worsens during the adverse event reporting period.

- Procedures

Diagnostic and therapeutic procedures, such as surgery, should not be reported as adverse events, while the medical condition for which the procedure was performed should be reported if it meets the definition of an adverse event. For example, an appendectomy performed for an acute appendicitis occurring during the adverse event reporting period should not be reported as adverse event; while “acute appendicitis” is to be reported as adverse event. If a patient undergoes a surgical procedure that was planned prior to entry into the trial, and surgery is not performed due to a worsening of a baseline condition, this baseline condition should not be reported as an adverse event.

- Symptoms of Targeted Disease

Tumour-related signs and symptoms will be followed at each visit. Although a measure of efficacy, these will always be reported as pre-existing conditions at baseline and during treatment only if they meet the definition of adverse event.

For all adverse events the Investigator will be asked to assess its relationship with each IMP or with the study treatment.

## 15.6 Causality assessment and Grading of Adverse Event Severity

The assessment of relationship to study drug and relevant WHO definitions will be done according to the following causality scale:

- ✓ Certain: A clinical event, including laboratory test abnormality, occurring in a plausible time relationship to drug administration, and which cannot be explained by concurrent disease or other drugs or chemicals. The response to withdrawal of the drug (dechallenge) should be clinically plausible. The event must be definitive pharmacologically or phenomenologically, using a satisfactory rechallenge procedure if necessary
- ✓ Probable: A clinical event, including laboratory test abnormality, with a reasonable time sequence to administration of the drug, unlikely to be attributed to concurrent disease or other drugs or chemicals, and which follows a clinically reasonable response on withdrawal (dechallenge). Rechallenge information is not required to fulfil this definition.
- ✓ Possible: A clinical event, laboratory test abnormality, with a reasonable time sequence to administration of the drug, but which could also be explained by concurrent disease or other drugs or chemicals. Information on drug withdrawal may be lacking or unclear
- ✓ Unlikely: A clinical event, laboratory test abnormality, with a temporal relationship to drug administration which makes a causal relationship improbable, and in which other drugs, chemicals or underlying disease provide plausible explanations

Severity grading of adverse events and pre-existing conditions will be evaluated according to the National Cancer Institute (NCI) Common Toxicity Criteria (*CTCAE) V4.03* (see Appendix 3).

Note the distinction between the gravity and the intensity of an adverse event. Severe is a measure of intensity; thus, a severe reaction is not necessarily a serious reaction. For example, a headache may be severe in intensity but would not be classified as serious unless it meets one of the criteria for serious events listed above.

## 15.7 Exposure in Utero

If any trial patient becomes or is found to be pregnant while receiving the study drug or within 90 days of last IMP dose, the investigator submits this information following the same procedure as for SAEs. This must be done irrespective of whether an adverse event has occurred and within 24 hours of awareness of the pregnancy. The information submitted should include the anticipated date of delivery (see below for information related to induce termination of pregnancy).

The investigator will follow the patient until completion of the pregnancy or until pregnancy termination (i.e., induced abortion) and then notify the Safety Desk of the outcome within 5 days or as specified below. The investigator will provide this information as a follow up to the initial exposure in utero notification. The reason(s) for an induced abortion must be specified.

If the outcome of the pregnancy meets the criteria for immediate classification as a serious adverse event (i.e., spontaneous abortion, stillbirth, neonatal death, or congenital anomaly [including that in an aborted fetus]), the investigator should follow procedures for reporting serious adverse events, i.e., report the event to the Safety Desk (mlorini@asst-pg23.it).

Additional specification of pregnancy outcomes that are classified as serious adverse events:

● "Spontaneous abortion" includes miscarriage and missed abortion.

● All neonatal deaths that occur within 1 month of birth should be reported, without regard to causality, as serious adverse events. In addition, any infant death after 1 month that the investigator assesses as possibly related to the in utero exposure to the investigational medication should also be reported

## 15.8 Overdose

Overdose (accidental or intentional) must be handled following the SAE procedures. This includes reports related to drug intake with suicidal intentions and consequent drug overdose.

Overdose reporting even not associated with adverse reactions shall be anyhow reported immediately to the Safety Desk, using the most rapid type of communication (phone, e-mail).

### 15.9 Follow-up of Unresolved Adverse Events

All adverse events should be followed until the end of the study. Drug-related and serious adverse events ongoing at the end of this observation period must be recorded until they are resolved or the investigator assesses them as chronic or the subject is lost to follow-up or starts a new anti-cancer treatment, whichever occurs earlier.

# 16 EFFICACY ASSESSMENTS

## 16.1 Definition of efficacy parameters

Absolute CD3+ count at +90 days after infusion in relation to the absolute number of BET cell infused and its composition (in terms of T-cell subsets and NK cells). OBD of BET will be defined as the absolute number of BET cell that will allow a CD3+ count of $\geq$ 600 x$10^6$/L at +90 days after infusion.

# 17 STATISTICAL METHODS

## 17.1 Sample size calculation

The study will be conducted according to an accelerated titration dose escalation rule to evaluate the MTD (Simon et al., 1997). Cohorts of one new patient per dose level will be used during the initial accelerated stage of the trial, if the number of BET generated is sufficient. When the first instance of DLT is observed or the second instance of any moderate (grade 2) toxicity, the cohort will be expanded for current dose level and reverted to use of a classic 3+3 design for all further cohorts. After the dose escalation phase, an expansion cohort is planned to further characterize the safety and the immune reconstitution potential of BET cell infusion. The estimated sample size for the expansion cohort is based on the consideration that BET infusion will allow an immune reconstitution ($\geq$600 CD3+ cells x$10^6$/L) after 3 months in at least 50% of the patients, given that without BET support is expected to be recorded in less than 10% in the historical non-treated cohort (see Section 4.4). With a 1-side test for a proportion, nine evaluable patients will provide 90% power to detect a statistically significant difference with an alpha error of 0.05.

With this design, it is expected to enroll 4-24 patients for the dose escalation and 9 patients for the cohort expansion for a total of 13-33 patients. Assuming a drop off rate of less than 5% due to ineligibility for poor clinical response after the third cycle, BET cell contamination by CD19+ cells, and/or inadequate BET cell production for the assigned dose level (during dose escalation) additional 1-2 patients may be enrolled for a total number of up to 35 patients.

## 17.2 Study Population

The populations used for analysis will include the following:

- **Enrolled patients**: this population will include all subjects who are enrolled, regardless of whether subjects receive the study drug or not. This population will be evaluated in the analysis of patients' disposition
- **Treated patients**: the treated patient population consists of all enrolled subjects who actually received BET infusion. This is the patient population used for all safety analyses (intention-to-treat analysis)
- **Endpoint-evaluable population**: patients who receive at least 1 dose of study drug have measurable endpoint parameters at baseline, and one post-baseline assessment, will be used for endpoint analyses.

## 17.3 Analysis

Summary statistics and analyses will be overall and by dose level where appropriate. Characteristics of patients will be summarized by means of cross-tabulations (categorical variables), quantiles (median etc; for ordinal factors) or by means of standard positional and variation parameters (mean, standard deviation; for continuous variables). Non-parametric tests will be applied, in univariate analysis, for comparisons between groups (Chi-Squared and Fisher Exact test for difference in terms of categorical variables or response rate, Mann-Whitney and Kruskal-Wallis test for difference in terms of continuous variables).

### 17.3.1 Study Conduct and Subject Disposition

All enrolled patients will be tabulated and listed. The number of patients withdrawing from the study, not meeting the eligibility criteria, and who are considered protocol violators will also be described.

### 17.3.2 Baseline Characteristics and treatment Group Comparability

Patient characteristics at study entry will be summarized in frequency tables and descriptive statistics will be provided for quantitative variables.

### 17.3.3 Treatment Analysis

The number of cells administered, as well as reasons for deviation from planned therapy and overall duration of treatment will be described.

### 17.3.4 Safety analysis

Safety and tolerability is the primary objective of the study. The incidence of DLT will be tabulated for each dose group. In addition, to assess the relationship between toxicities and BET cell dose, the preferred term of individual toxicities will be summarized by their frequency and intensity for each dose group. The DLT-evaluable population will be used for the analysis of DLT. AEs will be coded and displayed by the highest level term (preferred term) of the Medical Dictionary for Regulatory Activities (MedDRA) and graded according to NCI CTCAE version 4.03. The analysis will address all events that are recorded as occurring on treatment and whose onset date is posterior to the date of first treatment administration. AEs will be tabulated in the following categories:

- Treatment-emergent AEs
- Drug-related treatment-emergent AEs
- Grade 3 or higher treatment-emergent AEs
- Grade 3 or higher drug-related treatment-emergent AEs
- The most commonly reported treatment-emergent AEs (ie, those events reported by 10% of all patients)
- SAEs

A listing of treatment-emergent AEs resulting in study drug discontinuation will be provided.

### 17.3.5 Efficacy analysis

Immune reconstitution and evaluation of BET cell production are secondary efficacy objectives of the study. All analysis will be performed in the endpoint-evaluable population, including dose escalation and dose expansion patients. The following parameters will be tabulated:

- Absolute number of BET expanded and their composition (in terms of T-cell subsets and NK cells) in relation to absolute number of starting CD3+ and CD19+ lymphocytes
- Absolute CD3+ count +90 days after infusion in relation to the absolute number of BET cell infused and its composition (in terms of T-cell subsets and NK cells). OBD of BET will be defined as the absolute number of BET cell that will allow a CD3+ count of $\geq$ 600 x$10^6$/L at +90 days after infusion in at least 70% of the patients treated during dose expansion.
- Absolute numbers of B, T, and NK cells reconstitution at +0 (4 hours), +30, +90 and +180 days after infusion and its correlation with BET cell infused and its composition (in terms of T-cell subsets and NK cells)
- Evaluation of ex vivo transfer of anti-viral immunity in terms of tetramer-based quantification of CMV-specific CD8+ T lymphocytes at +0 (4 hours), +30, +90 and +180 days after infusion (this will be done only for CMV positive patients for whom CMV specific tetramers stain positive in starting peripheral blood or BET)

Data will be reported as number with percentage or median with range. All efficacy endpoints will be studied according to baseline characteristics. Pearson correlation coefficients will be calculated to find correlations between variables. Regression models will be performed in univariate and multivariate context to evaluate factors related to immune reconstitution and BET cell production.

# 18 QUALITY CONTROL AND QUALITY ASSURANCE

The Clinical Trial Quality Team (CTQT) has been established at ASST-Papa Giovanni XXIII for the management of clinical study and will handle all quality aspect of this trial as well as its management according to Italian law, GCPs and main rules concerning clinical trials.

## 18.1 Monitoring

Monitoring visits to the trial site will be made during the trial by a qualified monitor to verify that the trial is conducted according to study protocol, GCP principles and regulatory requirements. The monitor will verify the accurate and complete recording of data on CRFs, source documents, Investigators File and drug accountability records.

The investigator/institution guarantees direct access to source documents of the study patients and to any other trial related documentation.

It is important that the investigator(s) and/or their relevant personnel are available during the monitoring visits.

## 18.2 Auditing

Personnel designated of CTQT may conduct an audit at site. The investigator will be informed if an audit is to take place and advised as to the scope of the audit.
Representative of regulatory agency may also conduct an inspection of the study. If informed of such an inspection, the Investigator should notify CTQT immediately. The investigator will ensure that the auditors/ inspectors have access to the clinical supply, study site facilities, source documents and all study files.

## 18.3 Laboratory Requirements

For laboratories handling clinical laboratory samples, the accreditation certificate and laboratory normal units and ranges must be provided to the CTQT and must be updated as needed.

# 19 DATA HANDLING AND RECORD KEEPING

## 19.1 Case Report Form (CRF)

An electronic Case Report Form will be completed for each enrolled subject. The language used must be English. The completed original Case Report Forms are the sole property of the Sponsor and should not be made available in any form to third parties, except for authorized representatives of appropriate regulatory authorities, without written permission from Sponsor.

The Investigator or an authorized staff member (medically qualified) has the responsibility to ensure completion and to review and sign all Case Report Forms.

However, the Investigator has final personal responsibility for the accuracy and authenticity of all clinical and laboratory data entered on the Case Report Form.

Subject source documents are the hospital subject records maintained at the study site. In case where the source documents are the hospital chart, the information collected on the Case Report Form must match with those charts. In some case a portion of the source documents are not the hospital subject records. The investigator and Sponsor must agree which items will be recorded in the source documents and for which items the Case Report Form will stand as the source document. This must be stated in the "Data Location List" (filed in the Investigator File). One copy of this document should be remitted to the CTQT for filing into the Trial Master File.

## 19.2 Data Handling

Data Management, including analysis, will be carried out by the CTQT. Authorized investigators must enter the information required by the protocol into the electronic Case Report Forms (e-CRFs), through an electronic data capture system. The web portal interconnects the clinical database of the study where data will be recorded and saved.

The e-CRFs can be saved as draft documents while the Investigators waiting for instrumental or laboratory data; updates/changes to data entered can be done after the completion of e-CRF, but any correction must be justified and documented. All changes to e-form and data recorded will be tracked in the electronic database appropriately and report listing all data corrections is generated and updated authomatically, so that it can be used by the monitor for the resolution of queries.

Medical terms are coded according to the MedDRA dictionary. Data cleaning will include both visual and computer-driven procedures in order to minimize logical inconsistencies and errors within the collected data. The data are checked for completeness, accuracy and consistency. Data will be analyzed using STATA.

## 19.3 Record Retention

To enable evaluation and/or audits and/or regulatory authority inspections, the Investigator agrees to keep records, including the identity of all participating subjects ("Subject identification code

list”), all original signed informed consent forms, copies of all case report forms, source documents, detailed records of treatment disposition as well as the documentation included in the Investigator Trial File for seven years after editing the Final Study Report at conclusion of the study, according to Italian rules for non-commercial trials.

# 20 ETHICAL CONSIDERATION

## 20.1 Institutional Review Board(IRB)/ Independent Ethics Committee (IEC) and Competent Authority (CA)

Before initiating the trial, the Sponsor will have written favourable opinion from the IRB/IEC and CA for the trial conduction. All the correspondence with the IRB/IEC and CA should be retained in the Trial Master File and Investigator File at experimental unit.

Before implementing any protocol amendment, the IRB/IRC/CA written approval must be obtained. The only circumstance in which an amendment may be initiated prior to IRB/IEC approval is where the change is necessary to eliminate apparent immediate hazards to the subjects. In that event, the IRB/IEC/CA must be notified in writing asap.

It is responsibility of ASST-Papa Giovanni XXIII to provide the Investigator with the Health Authority approval where needed to implement a trial.

## 20.2 Ethical conduct of the trial

The trial will be performed in accordance with International Conference on Harmonization Good Clinical Practice guidelines [integrated addendum to GCP E6 (R2)], the Declaration of Helsinki and applicable local regulatory requirements and laws.

## 20.3 Informed Consent

It is the responsibility of the investigator to give each patient (or the patient acceptable representative) full and adequate verbal and written information regarding the objective and procedures of the trial and the possible risks involved. Patient candidate to be enrolled, will be explained in non-technical language about the purposes and the modalities of the study, the expected benefits and the probable discomfort, the procedures for management of adverse events, the insurance management and the procedures for reimbursement of the damage, and the possibility to leave the study at any time without motivation.

Investigators will hand, read and explain to the subject any information documents, as well as the implications of signing the informed consent, the participation and the processing of personal data.

Any further request for information will be met at any time during the study (will be given telephone numbers of the investigator); the clinical and personal data will be managed and protected, respecting confidentiality, under the terms of the law and within the limits indicated by the information given to the patient.

In this study Subjects unable to understand and to autonomously sign an informed consent, nor any minor will not be enrolled.

Written subject information must be approved by IRB/IEC and CA and must be given to each patient before any trial-related procedure is undertaken.

It is responsibility of the investigator to obtain informed consent signed and dated by the patient and by the medical person conducting the informed consent discussion, prior to undertaken any trial-related procedure. One copy of the signed and dated Informed Consent Form should be given to the patient. The originally signed document should be archived in the confidential section of the Investigator File.

The approved patient information sheet must not be changed without prior approval by the IRB/IEC and CA.

When new study information arises during the study, the patients still on treatment must be informed and a new Informed Consent form or an addendum to the already signed Informed Consent form must be signed and dated by the patients.

If a patient becomes incompetent during the course of a trial where it was not anticipated, legally acceptable representative authorization should be obtained for a subject's continued participation

# 21. STUDY DISCONTINUATION CRITERIA

The Sponsor reserves the right to discontinue the trial prior to inclusion of the intended number of subjects, but intends only to exercise this right for valid scientific or administrative reasons. After such a decision, the Investigator must contact all participating subjects within a time

period. In any case, for subjects on treatment, study product will be guaranteed until the subjects will benefit from these treatments. All study materials must be collected and all electronic case report forms completed to the greatest extent possible. The Sponsor will be responsible for informing IRBs and/or ECs of the early termination of the trial.

# 22 LIABILITY AND INSURANCE

Clinicians will work according to Decreto Ministeriale (DM) of 17/12/2004, published on GU n. 43 at 22/02/2005, that regulates non-commercial clinical trials, and has been imposed to improve clinical practice. Hence, the patients will be treated according to best clinical standards within their rights as citizens under the Sistema Sanitario Nazionale. This trial will be covered by insurance policy according to DM of July 14, 2009.

# 23 CONFIDENTIALITY OF INFORMATION AND PUBLICATION OF RESULTs

In order to maintain patient privacy, all data records, study drug accountability records, study reports and will be treated anonymously by coordinating data center, where no personal data to identify patient will be recorded. Patients will be identified in the study by the unique progressive number UPN.

Combined identification codes/passwords before access is granted to the computerized system and at the start of a data entry session. SSL connection is used to assure secure data transactions.

To guarantee the secrecy of the data, but also to avoid manipulation and loss of data, precautionary action (hardware and software) are taken.

In particular:

1. Access to data collected from the participating centres is reserved only to authorized members of the Sponsor (CTQT)
2. The data-collection network is protected by a firewall
3. The internet connection is encrypted with a digital certificate (SSL technology)
4. The database is located on a server that is protected with a password, that is changed periodically

5. Access to the database is protected with a password and is only accessible by responsible persons of Sponsor (CTQT)
6. Periodical back-ups will guarantee secure copies, to allow retrieval of both stored data and the data- collection system
7. The patient is registered and identifiable with a code, to guarantee anonymity

All investigators and members of CTQT participating to the trial agrees to keep in confidence all the results obtained from the study. Such information shall not be disclosed to third parties without prior written permission from Principal Investigator, except to regulatory authority(ies), when requested

At trial conclusion, when final analyses have been performed, the Principal Investigator will have the rights to use the data arising from the trial, for congress communications and scientific publications of the study results. The Principal Investigator of the study will have full access to all data of the study and has full and total responsibility of:

- The preparation of the manuscript(s) and of collected data in this study
- The final decision on the number, order and the names of the contributing authors

Investigators and other personnel participating to the study design will be listed as co-author.

# 24 REFERENCES

Ardeshna, K. M., Smith, P., Norton, A., Hancock, B. W., Hoskin, P. J., MacLennan, K. A., Marcus, R. E., Jelliffe, A., Vaughan, G., Hudson, and Linch, D. C. (2003). Long-term effect of a watch and wait policy versus immediate systemic treatment for asymptomatic advanced-stage non-Hodgkin lymphoma: a randomised controlled trial. Lancet *362*, 516-522.

Berdeja, J. G., Hess, A., Lucas, D. M., O'Donnell, P., Ambinder, R. F., Diehl, L. F., Carter-Brookins, D., Newton, S., and Flinn, I. W. (2007). Systemic interleukin-2 and adoptive transfer of lymphokine-activated killer cells improves antibody-dependent cellular cytotoxicity in patients with relapsed B-cell lymphoma treated with rituximab. Clin Cancer Res *13*, 2392-2399.

Bomberger, C., Singh-Jairam, M., Rodey, G., Guerriero, A., Yeager, A. M., Fleming, W. H., Holland, H. K., and Waller, E. K. (1998). Lymphoid reconstitution after autologous PBSC transplantation with FACS-sorted CD34+ hematopoietic progenitors. Blood *91*, 2588-2600.

Brice, P., Bastion, Y., Lepage, E., Brousse, N., Haioun, C., Moreau, P., Straetmans, N., Tilly, H., Tabah, I., and Solal-Celigny, P. (1997). Comparison in low-tumor-burden follicular lymphomas between an initial no-treatment policy, prednimustine, or interferon alfa: a randomized study from the Groupe d'Etude des Lymphomes Folliculaires. Groupe d'Etude des Lymphomes de l'Adulte. J Clin Oncol *15*, 1110-1117.

Cheson, B. D., Fisher, R. I., Barrington, S. F., Cavalli, F., Schwartz, L. H., Zucca, E., and Lister, T. A. (2014). Recommendations for initial evaluation, staging, and response assessment of Hodgkin and non-Hodgkin lymphoma: the Lugano classification. J Clin Oncol *32*, 3059-3068.

CLL Trialists' Collaborative Group (1999). Chemotherapeutic options in chronic lymphocytic leukemia: a meta-analysis of the randomized trials. CLL Trialists' Collaborative Group. J Natl Cancer Inst *91*, 861-868.

Cockcroft, D. W., and Gault, M. H. (1976). Prediction of creatinine clearance from serum creatinine. Nephron *16*, 31-41.

Darwish, M., Bond, M., Hellriegel, E., Robertson, P., Jr., and Chovan, J. P. (2015). Pharmacokinetic and pharmacodynamic profile of bendamustine and its metabolites. Cancer Chemother Pharmacol *75*, 1143-1154.

Dreier, T., Lorenczewski, G., Brandl, C., Hoffmann, P., Syring, U., Hanakam, F., Kufer, P., Riethmuller, G., Bargou, R., and Baeuerle, P. A. (2002). Extremely potent, rapid and costimulation-independent cytotoxic T-cell response against lymphoma cells catalyzed by a single-chain bispecific antibody. Int J Cancer *100*, 690-697.

Eichhorst, B., Fink, A. M., Bahlo, J., Busch, R., Kovacs, G., Maurer, C., Lange, E., Koppler, H., Kiehl, M., Sokler, M., *et al.* (2016). First-line chemoimmunotherapy with bendamustine and rituximab versus fludarabine, cyclophosphamide, and rituximab in patients with advanced chronic lymphocytic leukaemia (CLL10): an international, open-label, randomised, phase 3, non-inferiority trial. Lancet Oncol.

Ferlay, J., Steliarova-Foucher, E., Lortet-Tieulent, J., Rosso, S., Coebergh, J. W., Comber, H., Forman, D., and Bray, F. (2013). Cancer incidence and mortality patterns in Europe: estimates for 40 countries in 2012. Eur J Cancer *49*, 1374-1403.

Flinn, I. W., van der Jagt, R., Kahl, B. S., Wood, P., Hawkins, T. E., Macdonald, D., Hertzberg, M., Kwan, Y. L., Simpson, D., Craig, M., *et al.* (2014). Randomized trial of bendamustine-rituximab or R-CHOP/R-CVP in first-line treatment of indolent NHL or MCL: the BRIGHT study. Blood *123*, 2944-2952.

Garcia Munoz, R., Izquierdo-Gil, A., Munoz, A., Roldan-Galiacho, V., Rabasa, P., and Panizo, C. (2014). Lymphocyte recovery is impaired in patients with chronic lymphocytic leukemia and indolent non-Hodgkin lymphomas treated with bendamustine plus rituximab. Ann Hematol *93*, 1879-1887.

Garlie, N. K., LeFever, A. V., Siebenlist, R. E., Levine, B. L., June, C. H., and Lum, L. G. (1999). T cells coactivated with immobilized anti-CD3 and anti-CD28 as potential immunotherapy for cancer. J Immunother *22*, 336-345.

Golay, J., D'Amico, A., Borleri, G., Bonzi, M., Valgardsdottir, R., Alzani, R., Cribioli, S., Albanese, C., Pesenti, E., Finazzi, M. C., *et al.* (2014). A novel method using blinatumomab for efficient, clinical-grade expansion of polyclonal T cells for adoptive immunotherapy. J Immunol *193*, 4739-4747.

Gribben, J. G. (2007). How I treat indolent lymphoma. Blood *109*, 4617-4626.

Grupp, S. A., Kalos, M., Barrett, D., Aplenc, R., Porter, D. L., Rheingold, S. R., Teachey, D. T., Chew, A., Hauck, B., Wright, J. F., *et al.* (2013). Chimeric antigen receptor-modified T cells for acute lymphoid leukemia. N Engl J Med *368*, 1509-1518.

Hallek, M., Cheson, B. D., Catovsky, D., Caligaris-Cappio, F., Dighiero, G., Dohner, H., Hillmen, P., Keating, M. J., Montserrat, E., Rai, K. R., and Kipps, T. J. (2008). Guidelines for the diagnosis and treatment of chronic lymphocytic leukemia: a report from the International Workshop on Chronic Lymphocytic Leukemia updating the National Cancer Institute-Working Group 1996 guidelines. Blood *111*, 5446-5456.

Introna, M., Borleri, G., Conti, E., Franceschetti, M., Barbui, A. M., Broady, R., Dander, E., Gaipa, G., D'Amico, G., Biagi, E., *et al.* (2007). Repeated infusions of donor-derived cytokine-induced killer cells in patients relapsing after allogeneic stem cell transplantation: a phase I study. Haematologica *92*, 952-959.

Introna, M., Pievani, A., Borleri, G., Capelli, C., Algarotti, A., Mico, C., Grassi, A., Oldani, E., Golay, J., and Rambaldi, A. (2010). Feasibility and safety of adoptive immunotherapy with CIK cells after cord blood transplantation. Biol Blood Marrow Transplant *16*, 1603-1607.

Kalamasz, D., Long, S. A., Taniguchi, R., Buckner, J. H., Berenson, R. J., and Bonyhadi, M. (2004). Optimization of human T-cell expansion ex vivo using magnetic beads conjugated with anti-CD3 and Anti-CD28 antibodies. J Immunother *27*, 405-418.

Kochenderfer, J. N., Dudley, M. E., Feldman, S. A., Wilson, W. H., Spaner, D. E., Maric, I., Stetler-Stevenson, M., Phan, G. Q., Hughes, M. S., Sherry, R. M., *et al.* (2012). B-cell depletion and remissions of malignancy along with cytokine-associated toxicity in a clinical trial of anti-CD19 chimeric-antigen-receptor-transduced T cells. Blood *119*, 2709-2720.

Levine, B. L., Bernstein, W. B., Connors, M., Craighead, N., Lindsten, T., Thompson, C. B., and June, C. H. (1997). Effects of CD28 costimulation on long-term proliferation of CD4+ T cells in the absence of exogenous feeder cells. J Immunol *159*, 5921-5930.

Levine, B. L., Cotte, J., Small, C. C., Carroll, R. G., Riley, J. L., Bernstein, W. B., Van Epps, D. E., Hardwick, R. A., and June, C. H. (1998). Large-scale production of CD4+ T cells from HIV-1-infected donors after CD3/CD28 costimulation. J Hematother *7*, 437-448.

Loffler, A., Gruen, M., Wuchter, C., Schriever, F., Kufer, P., Dreier, T., Hanakam, F., Baeuerle, P. A., Bommert, K., Karawajew, L., *et al.* (2003). Efficient elimination of chronic lymphocytic

leukaemia B cells by autologous T cells with a bispecific anti-CD19/anti-CD3 single-chain antibody construct. Leukemia *17*, 900-909.

Long-Boyle, J. R., Green, K. G., Brunstein, C. G., Cao, Q., Rogosheske, J., Weisdorf, D. J., Miller, J. S., Wagner, J. E., McGlave, P. B., and Jacobson, P. A. (2011). High fludarabine exposure and relationship with treatment-related mortality after nonmyeloablative hematopoietic cell transplantation. Bone Marrow Transplant *46*, 20-26.

Mackall, C. L. (1999). T-cell immunodeficiency following cytotoxic antineoplastic therapy: a review. Oncologist *4*, 370-378.

Maus, M. V., Thomas, A. K., Leonard, D. G., Allman, D., Addya, K., Schlienger, K., Riley, J. L., and June, C. H. (2002). Ex vivo expansion of polyclonal and antigen-specific cytotoxic T lymphocytes by artificial APCs expressing ligands for the T-cell receptor, CD28 and 4-1BB. Nat Biotechnol *20*, 143-148.

Nagorsen, D., and Baeuerle, P. A. (2011). Immunomodulatory therapy of cancer with T cell-engaging BiTE antibody blinatumomab. Exp Cell Res *317*, 1255-1260.

Rapoport, A. P., Stadtmauer, E. A., Aqui, N., Badros, A., Cotte, J., Chrisley, L., Veloso, E., Zheng, Z., Westphal, S., Mair, R., *et al.* (2005). Restoration of immunity in lymphopenic individuals with cancer by vaccination and adoptive T-cell transfer. Nat Med *11*, 1230-1237.

Rapoport, A. P., Stadtmauer, E. A., Aqui, N., Vogl, D., Chew, A., Fang, H. B., Janofsky, S., Yager, K., Veloso, E., Zheng, Z., *et al.* (2009). Rapid immune recovery and graft-versus-host disease-like engraftment syndrome following adoptive transfer of Costimulated autologous T cells. Clin Cancer Res *15*, 4499-4507.

Robak, T., Lech-Maranda, E., Korycka, A., and Robak, E. (2006). Purine nucleoside analogs as immunosuppressive and antineoplastic agents: mechanism of action and clinical activity. Curr Med Chem *13*, 3165-3189.

Robak, T., Stilgenbauer, S., and Tedeschi, A. (2016). Front-line treatment of CLL in the era of novel agents. Cancer Treat Rev *53*, 70-78.

Rummel, M. J., Niederle, N., Maschmeyer, G., Banat, G. A., von Grunhagen, U., Losem, C., Kofahl-Krause, D., Heil, G., Welslau, M., Balser, C., *et al.* (2013). Bendamustine plus rituximab versus CHOP plus rituximab as first-line treatment for patients with indolent and mantle-cell

lymphomas: an open-label, multicentre, randomised, phase 3 non-inferiority trial. Lancet *381*, 1203-1210.

Saito, H., Maruyama, D., Maeshima, A. M., Makita, S., Kitahara, H., Miyamoto, K., Fukuhara, S., Munakata, W., Suzuki, T., Kobayashi, Y., *et al.* (2015). Prolonged lymphocytopenia after bendamustine therapy in patients with relapsed or refractory indolent B-cell and mantle cell lymphoma. Blood Cancer J *5*, e362.

Solal-Celigny, P., Bellei, M., Marcheselli, L., Pesce, E. A., Pileri, S., McLaughlin, P., Luminari, S., Pro, B., Montoto, S., Ferreri, A. J., *et al.* (2012). Watchful waiting in low-tumor burden follicular lymphoma in the rituximab era: results of an F2-study database. J Clin Oncol *30*, 3848-3853.

Stadtmauer, E. A., Vogl, D. T., Luning Prak, E., Boyer, J., Aqui, N. A., Rapoport, A. P., McDonald, K. R., Hou, X., Murphy, H., Bhagat, R., *et al.* (2011). Transfer of influenza vaccine-primed costimulated autologous T cells after stem cell transplantation for multiple myeloma leads to reconstitution of influenza immunity: results of a randomized clinical trial. Blood *117*, 63-71.

Swerdlow, S. H., Campo, E., Pileri, S. A., Harris, N. L., Stein, H., Siebert, R., Advani, R., Ghielmini, M., Salles, G. A., Zelenetz, A. D., and Jaffe, E. S. (2016). The 2016 revision of the World Health Organization classification of lymphoid neoplasms. Blood *127*, 2375-2390.

Wong, R., Pepper, C., Brennan, P., Nagorsen, D., Man, S., and Fegan, C. (2013). Blinatumomab induces autologous T-cell killing of chronic lymphocytic leukemia cells. Haematologica *98*, 1930-1938.

Ysebaert, L., Gross, E., Kuhlein, E., Blanc, A., Corre, J., Fournie, J. J., Laurent, G., and Quillet-Mary, A. (2010). Immune recovery after fludarabine-cyclophosphamide-rituximab treatment in B-chronic lymphocytic leukemia: implication for maintenance immunotherapy. Leukemia *24*, 1310-1316.

# 25 APPENDICES

Appendix A: ECOG performance status scale

Appendix B: Creatinine Clearance Calculation: Cockcroft-Gault Equation

Appendix C: Common Terminology Criteria for Adverse Events

Appendix D: World Medical Association Declaration of Helsinki

# Appendix A - ECOG Performance Status Scale

| ECOG PERFORMANCE STATUS* | |
|---|---|
| **Grade** | **ECOG** |
| 0 | Fully active, able to carry on all pre-disease performance without restriction |
| 1 | Restricted in physically strenuous activity but ambulatory and able to carry out work of a light or sedentary nature, e.g., light house work, office work |
| 2 | Ambulatory and capable of all selfcare but unable to carry out any work activities. Up and about more than 50% of waking hours |
| 3 | Capable of only limited selfcare, confined to bed or chair more than 50% of waking hours |
| 4 | Completely disabled. Cannot carry on any selfcare. Totally confined to bed or chair |
| 5 | Dead |

* As published in Am. J. Clin. Oncol.: Oken, M.M., Creech, R.H., Tormey, D.C., Horton, J., Davis, T.E., McFadden, E.T., Carbone, P.P.: Toxicity And Response Criteria Of The Eastern Cooperative Oncology Group. Am J Clin Oncol 5:649-655, 1982.

# Appendix B: Creatinine Clearance Calculation: Cockcroft-Gault Equation

(Cockcroft and Gault, 1976)

Males:

$$Cl_{creat}\ (ml/min) = \frac{[140 - age\ (years)] \times [body\ weight\ (kg)]}{72 \times serum\ creatinine\ (mg/dl)}$$

Females:

$$Cl_{creat}\ (ml/min) = 0.85 \times (male\ value)$$

Available on-line at: http://www.mdcalc.com/creatinine-clearance-cockcroft-gault-equation

# Appendix C: Common Terminology Criteria for Adverse Events

In this study, adverse events and/or adverse drug reactions (with the exception of hematologic toxicity, see 9.2.1, Table 6) will be recorded according to the **Common Terminology Criteria for Adverse Events (CTCAE), version 4.0.** This reference is used as the standard grading scale for each single Adverse Event term.

The full version of the document (version 4.0) is available on the NCI website (http://evs.nci.nih.gov/ftp1/CTCAE/CTCAE_4.03_2010-06-14_QuickReference_5x7.pdf)

# Appendix D: World Medical Association Declaration of Helsinki

**WORLD MEDICAL ASSOCIATION DECLARATION OF HELSINKI**
Ethical Principles for Medical Research Involving Human Subjects

Adopted by the 18th WMA General Assembly, Helsinki, Finland, June 1964, and amended by the: 29th WMA General Assembly, Tokyo, Japan, October 1975, 35th WMA General Assembly, Venice, Italy, October 1983, 41st WMA General Assembly, Hong Kong, September 1989, 48th WMA General Assembly, Somerset West, Republic of South Africa, October 1996, 52nd WMA General Assembly, Edinburgh, Scotland, October 2000, 53th WMA General Assembly, Washington 2002 (Note of Clarification on paragraph 29 added), 55th WMA General Assembly, Tokyo 2004 (Note of Clarification on Paragraph 30 added), 59th WMA General Assembly, Seoul, October 2008

## A. INTRODUCTION

1. The World Medical Association (WMA) has developed the Declaration of Helsinki as a statement of ethical principles for medical research involving human subjects, including research on identifiable human material and data.

   The Declaration is intended to be read as a whole and each of its constituent paragraphs should not be applied without consideration of all other relevant paragraphs.
2. Although the Declaration is addressed primarily to physicians, the WMA encourages other participants in medical research involving human subjects to adopt these principles.
3. It is the duty of the physician to promote and safeguard the health of patients, including those who are involved in medical research. The physician's knowledge and conscience are dedicated to the fulfilment of this duty.
4. The Declaration of Geneva of the WMA binds the physician with the words, "The health of my patient will be my first consideration," and the International Code of Medical Ethics declares that, "A physician shall act in the patient's best interest when providing medical care."

5. Medical progress is based on research that ultimately must include studies involving human subjects. Populations that are underrepresented in medical research should be provided appropriate access to participation in research.
6. In medical research involving human subjects, the well-being of the individual research subject must take precedence over all other interests.
7. The primary purpose of medical research involving human subjects is to understand the causes, development and effects of diseases and improve preventive, diagnostic and therapeutic interventions (methods, procedures and treatments). Even the best current interventions must be evaluated continually through research for their safety, effectiveness, efficiency, accessibility and quality.
8. In medical practice and in medical research, most interventions involve risks and burdens.
9. Medical research is subject to ethical standards that promote respect for all human subjects and protect their health and rights. Some research populations are particularly vulnerable and need special protection. These include those who cannot give or refuse consent for themselves and those who may be vulnerable to coercion or undue influence.
10. Physicians should consider the ethical, legal and regulatory norms and standards for research involving human subjects in their own countries as well as applicable international norms and standards. No national or international ethical, legal or regulatory requirement should reduce or eliminate any of the protections for research subjects set forth in this Declaration.

## B. BASIC PRINCIPLES FOR ALL MEDICAL RESEARCH

11. It is the duty of physicians who participate in medical research to protect the life, health, dignity, integrity, right to self-determination, privacy, and confidentiality of personal information of research subjects.
12. Medical research involving human subjects must conform to generally accepted scientific principles, be based on a thorough knowledge of the scientific literature, other relevant sources of information, and adequate laboratory and, as appropriate, animal experimentation. The welfare of animals used for research must be respected.
13. Appropriate caution must be exercised in the conduct of medical research that may harm the environment.

14. The design and performance of each research study involving human subjects must be clearly described in a research protocol. The protocol should contain a statement of the ethical considerations involved and should indicate how the principles in this Declaration have been addressed. The protocol should include information regarding funding, sponsors, institutional affiliations, other potential conflicts of interest, incentives for subjects and provisions for treating and/or compensating subjects who are harmed as a consequence of participation in the research study. The protocol should describe arrangements for post-study access by study subjects to interventions identified as beneficial in the study or access to other appropriate care or benefits.
15. The research protocol must be submitted for consideration, comment, guidance and approval to a research ethics committee before the study begins. This committee must be independent of the researcher, the sponsor and any other undue influence. It must take into consideration the laws and regulations of the country or countries in which the research is to be performed as well as applicable international norms and standards but these must not be allowed to reduce or eliminate any of the protections for research subjects set forth in this Declaration. The committee must have the right to monitor ongoing studies. The researcher must provide monitoring information to the committee, especially information about any serious adverse events. No change to the protocol may be made without consideration and approval by the committee.
16. Medical research involving human subjects must be conducted only by individuals with the appropriate scientific training and qualifications. Research on patients or healthy volunteers requires the supervision of a competent and appropriately qualified physician or other health care professional. The responsibility for the protection of research subjects must always rest with the physician or other health care professional and never the research subjects, even though they have given consent.
17. Medical research involving a disadvantaged or vulnerable population or community is only justified if the research is responsive to the health needs and priorities of this population or community and if there is a reasonable likelihood that this population or community stands to benefit from the results of the research.

18. Every medical research study involving human subjects must be preceded by careful assessment of predictable risks and burdens to the individuals and communities involved in the research in comparison with foreseeable benefits to them and to other individuals or communities affected by the condition under investigation.
19. Every clinical trial must be registered in a publicly accessible database before recruitment of the first subject.
20. Physicians may not participate in a research study involving human subjects unless they are confident that the risks involved have been adequately assessed and can be satisfactorily managed. Physicians must immediately stop a study when the risks are found to outweigh the potential benefits or when there is conclusive proof of positive and beneficial results.
21. Medical research involving human subjects may only be conducted if the importance of the objective outweighs the inherent risks and burdens to the research subjects.
22. Participation by competent individuals as subjects in medical research must be voluntary. Although it may be appropriate to consult family members or community leaders, no competent individual may be enrolled in a research study unless he or she freely agrees.
23. Every precaution must be taken to protect the privacy of research subjects and the confidentiality of their personal information and to minimize the impact of the study on their physical, mental and social integrity.
24. In medical research involving competent human subjects, each potential subject must be adequately informed of the aims, methods, sources of funding, any possible conflicts of interest, institutional affiliations of the researcher, the anticipated benefits and potential risks of the study and the discomfort it may entail, and any other relevant aspects of the study. The potential subject must be informed of the right to refuse to participate in the study or to withdraw consent to participate at any time without reprisal. Special attention should be given to the specific information needs of individual potential subjects as well as to the methods used to deliver the information. After ensuring that the potential subject has understood the information, the physician or another appropriately qualified individual must then seek the potential subject's freely-given informed consent, preferably in writing. If the consent cannot be expressed in writing, the non-written consent must be formally documented and witnessed.

25. For medical research using identifiable human material or data, physicians must normally seek consent for the collection, analysis, storage and/or reuse. There may be situations where consent would be impossible or impractical to obtain for such research or would pose a threat to the validity of the research. In such situations the research may be done only after consideration and approval of a research ethics committee.
26. When seeking informed consent for participation in a research study the physician should be particularly cautious if the potential subject is in a dependent relationship with the physician or may consent under duress. In such situations the informed consent should be sought by an appropriately qualified individual who is completely independent of this relationship.
27. For a potential research subject who is incompetent, the physician must seek informed consent from the legally authorized representative. These individuals must not be included in a research study that has no likelihood of benefit for them unless it is intended to promote the health of the population represented by the potential subject, the research cannot instead be performed with competent persons, and the research entails only minimal risk and minimal burden.
28. When a potential research subject who is deemed incompetent is able to give assent to decisions about participation in research, the physician must seek that assent in addition to the consent of the legally authorized representative. The potential subject's dissent should be respected.
29. Research involving subjects who are physically or mentally incapable of giving consent,for example, unconscious patients, may be done only if the physical or mental condition that prevents giving informed consent is a necessary characteristic of the research population. In such circumstances the physician should seek informed consent from the legally authorized representative. If no such representative is available and if the research cannot be delayed, the study may proceed without informed consent provided that the specific reasons for involving subjects with a condition that renders them unable to give informed consent have been stated in the research protocol and the study has been approved by a research ethics committee. Consent to remain in the research should be obtained as soon as possible from the subject or a legally authorized representative.

30. Authors, editors and publishers all have ethical obligations with regard to the publication of the results of research. Authors have a duty to make publicly available the results of their research on human subjects and are accountable for the completeness and accuracy of their reports. They should adhere to accepted guidelines for ethical reporting. Negative and inconclusive as well as positive results should be published or otherwise made publicly available. Sources of funding, institutional affiliations and conflicts of interest should be declared in the publication. Reports of research not in accordance with the principles of this Declaration should not be accepted for publication.

## C. ADDITIONAL PRINCIPLES FOR MEDICAL RESEARCH COMBINED WITH MEDICAL CARE

1. The physician may combine medical research with medical care only to the extent that the research is justified by its potential preventive, diagnostic or therapeutic value and if the physician has good reason to believe that participation in the research study will not adversely affect the health of the patients who serve as research subjects.
2. The benefits, risks, burdens and effectiveness of a new intervention must be tested against those of the best current proven intervention, except in the following circumstances:

   *0 The use of placebo, or no treatment, is acceptable in studies where no current proven intervention exists; or

   *1 Where for compelling and scientifically sound methodological reasons the use of placebo is necessary to determine the efficacy or safety of an intervention and the patients who receive placebo or no treatment will not be subject to any risk of serious or irreversible harm. Extreme care must be taken to avoid abuse of this option.
3. At the conclusion of the study, patients entered into the study are entitled to be informed about the outcome of the study and to share any benefits that result from it, for example, access to interventions identified as beneficial in the study or to other appropriate care or benefits.

4. The physician must fully inform the patient which aspects of the care are related to the research. The refusal of a patient to participate in a study or the patient's decision to withdraw from the study must never interfere with the patient-physician relationship.
5. In the treatment of a patient, where proven interventions do not exist or have been ineffective, the physician, after seeking expert advice, with informed consent from the patient or a legally authorized representative, may use an unproven intervention if in the physician's judgement it offers hope of saving life, re-establishing health or alleviating suffering. Where possible, this intervention should be made the object of research, designed to evaluate its safety and efficacy. In all cases, new information should be recorded and, where appropriate, made publicly available.
